# Bromopyrrole Alkaloids from the Sponge *Agelas kosrae*

**DOI:** 10.3390/md16120513

**Published:** 2018-12-17

**Authors:** Oh-Seok Kwon, Donghwa Kim, Heegyu Kim, Yeon-Ju Lee, Hyi-Seung Lee, Chung J. Sim, Dong-Chan Oh, Sang Kook Lee, Ki-Bong Oh, Jongheon Shin

**Affiliations:** 1 Natural Products Research Institute, College of Pharmacy, Seoul National University, San 56-1, Sillim, Gwanak, Seoul 151-742, Korea; ideally225@snu.ac.kr (O.-S.K.); kdh9298@gmail.com (D.K.); dongchanoh@snu.ac.kr (D.-C.O.); sklee61@snu.ac.kr (S.K.L.); 2 Department of Agricultural Biotechnology, College of Agriculture and Life Science, Seoul National University, San 56-1, Sillim, Gwanak, Seoul 151-921, Korea; hqhqeori@snu.ac.kr; 3 Marine Natural Products Laboratory, Korea Institute of Ocean Science and Technology, P.O. Box 29, Seoul 425-600, Korea; yjlee@kiost.ac.kr (Y.-J.L.); hslee@kiost.ac.kr (H.-S.L.); 4 Department of Biological Science, College of Life Science and Nano Technology, Hannam University, 461-6 Jeonmin, Yuseong, Daejeon 305-811, Korea; cjsim@hnu.kr

**Keywords:** sceptrin, pyrrole-imidazole alkaloids, marine sponge, anti-angiogenesis, isocitrate lyase, bioactive marine natural products

## Abstract

Two new sceptrin derivatives (**1**,**2**) and eight structurally-related known bromopyrrole-bearing alkaloids were isolated from the tropical sponge *Agelas kosrae*. By a combination of spectroscopic methods, the new compounds, designated dioxysceptrin (**1**) and ageleste C (**2**), were determined to be structural analogs of each other that differ at the imidazole moiety. Dioxysceptrin was also found to exist as a mixture of α-amido epimers. The sceptrin alkaloids exhibited weak cytotoxicity against cancer cells. Compounds **1** and **2** also moderately exhibited anti-angiogenic and isocitrate lyase-inhibitory activities, respectively.

## 1. Introduction

Pyrrole–imidazole alkaloids are widely distributed in marine sponges [1,2,3,4]. (−)-Dibromophakellin and oroidin are well-known early examples of this class of compounds discovered in the sponges *Phakellia flabellata* and *Agelas oroides*, respectively, that were found almost 50 years ago [5,6,7,8]. Since then, more than 200 pyrrole–imidazoles have been found from a variety of tropical sponges, which makes these compounds an important class of sponge-derived small alkaloids [1,2,3,4]. Among the immense structural variations of these compounds, one particularly abundant form is the dimerization of the C_11_ oroidin skeleton, which is represented by sceptrin, a dipyrrole–diimidazole metabolite bearing a cyclobutane core from the sponge *Agelas sceptrum* [1,3,4,9]. According to a recent comprehensive review, sceptrin-type dimeric compounds account for approximately one-third of all pyrrole–imidazole alkaloids [1,3,4]. Inspired by their wide structural variations and significant bioactivities, such as anticancer, antifungal and antibacterial activities, numerous works have focused on their chemical synthesis and biosynthesis [1,3,4,10,11,12,13].

**Figure 1:**
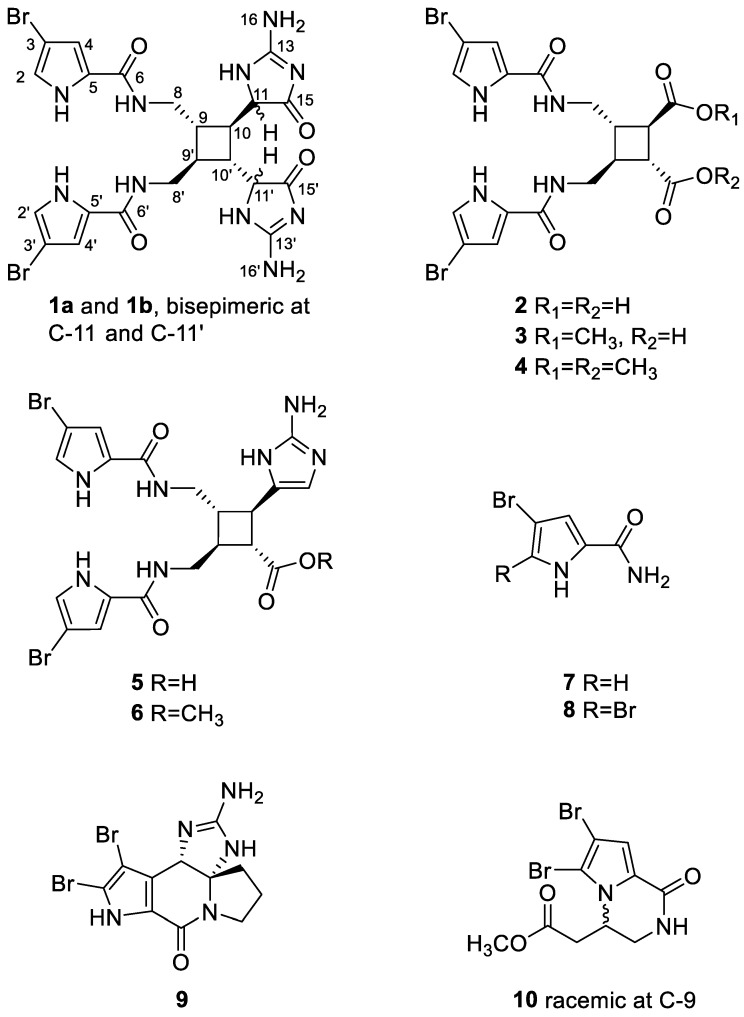
The structures of compounds **1**–**10**.

During the course of our search for bioactive metabolites from tropical sponges, we encountered the purple elongated sponge *Agelas kosrae* from Kosrae Island, the Federated States of Micronesia, and the organic extract of this sponge exhibited moderate cytotoxicity (IC_50_ 279 μg/mL) against the human chronic myeloid leukemia cell line-K562. Activity-guided separation of the extract employing diverse chromatographic methods led to the isolation of 10 bromopyrrole-bearing alkaloids similar to sceptrin and related structural classes including two new compounds. We report here the structural determination of dioxysceptrin (**1**) and ageleste C (**2**) by combinations of spectroscopic analyses (Figure 1). These sceptrin alkaloids exhibited weak cytotoxicity against six cancer cell lines (K562, A549, HCT116, MDA-MB-231, SNU628, SK-Hep-1). In addition, compounds **1** and **2** moderately exhibited anti-angiogenic and isocitrate lyase (ICL)-inhibitory activities, respectively.

## 2. Results and Discussion

The molecular formula of dioxysceptrin (**1**) was deduced as C_22_H_24_Br_2_N_10_O_4_ by HRFABMS analysis (*m/z* [M + H]^+^ 651.0432, calcd. 651.0427) aided by isotopic clusters in both positive (*m/z* 651.0/653.0/655.0) and negative ion modes (*m/z* 648.9/650.9/652.9) with intensities in a 1:2:1 ratio, indicating a dibrominated compound (Figure S13). However, an interesting phenomenon was found in the NMR spectra of this compound. That is, two sets of highly disproportionate signals existed in both the initial ^1^H and ^13^C NMR spectra. Then, during storage, the ratio between the intensities of these sets of peaks gradually reached equilibrium (from 6:1 to 1:1 according to the ^1^H NMR spectrum). Since several attempts to separate these compounds under various HPLC conditions were not successful, **1** was thought to be a mixture of either epimers or conformational isomers (**1a** and **1b**), and their structures were determined from the mixture.

In the ^13^C NMR spectrum of **1a**, three carbons at δ_C_ 174.3, 160.2 and 158.7 were thought to be amide carbonyl and/or guanidine carbons (Table 1). This interpretation was supported by the IR absorption bands at 1680 and 1635 cm^−1^. Four additional carbons at δ_C_ 126.6 (C), 121.3 (CH), 111.8 (CH), and 95.1 (C) in conjunction with the protons at δ_H_ 6.95 (1H, br s) and 6.84 (1H, br s) in the ^1^H NMR data were indicative of a substituted pyrrole moiety. The remaining carbons were the protonated ones in the more shielded region: δ_C_ 60.3 (CH), 41.9 (CH_2_), 38.2 (CH) and 37.1 (CH). A very similar set of carbon and proton signals was also found for **1b**.

**Figure 2:**
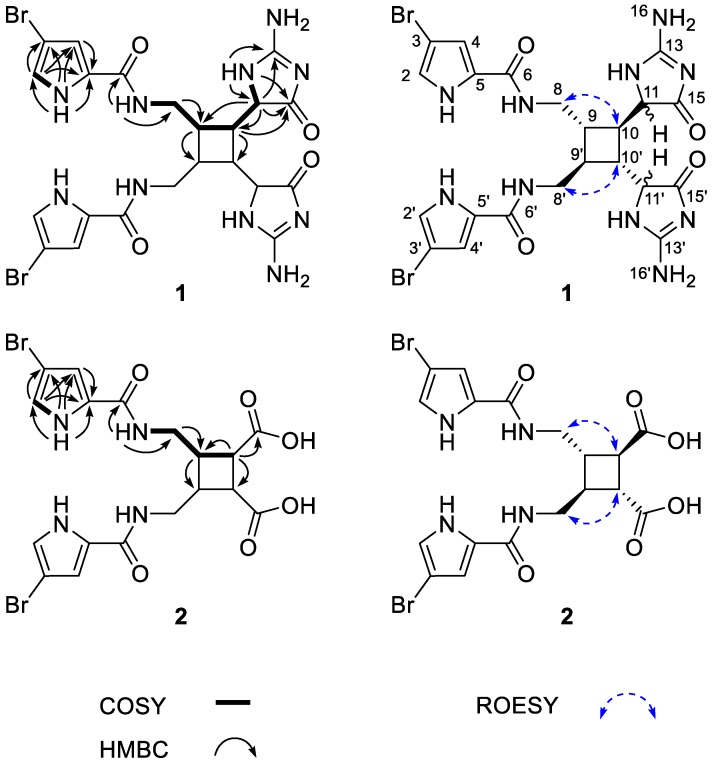
Key COSY (bold), HMBC (arrows), and ROESY (dashed arrows) correlations of compounds **1** and **2**.

Given this information, the planar structure of **1a** was determined by a combination of 2D NMR experiments. First, all of the carbons were matched to their attached protons by an HSQC experiment. Then, a direct connection was found between an aromatic proton (H-2) and an NH proton (NH-1) at δ_H_ 6.95 and 11.73, respectively, by a COSY experiment. The HMBC correlations of these protons and an additional proton at δ_H_ 6.84 (H-4) with the neighboring carbons readily identified a 2,4-disubstituted pyrrole moiety (1-NH-C-5) (Figure 2). The significant shielding of C-3 at δ_C_ 95.1 confirmed the attachment of bromine at this position. Similarly, although it was not directly found from the HMBC data, the shift of C-5 at δ_C_ 126.6 revealed the presence of a carbon substituent, possibly a carbonyl carbon, at this position.

The COSY data revealed a long proton spin system of alkyl protons with NH groups at both termini (7-NH-12-NH), and this assignment was supported by several HMBC correlations among the carbons and protons in this moiety. An amide linkage was found between this group and the previously identified bromopyrrole by an HMBC correlation between 6-NH and C-6 (δ_C_ 126.6). Similarly, a guanidine carbon and a carbonyl carbon were placed at C-13 and C-15, respectively, at the other terminus by a series of HMBC correlations: H-10/C-15, H-11/C-13 and C-15, and 12-NH/C-13 and C-15. Although it was not directly found from the 2D NMR data, the characteristic chemical shifts of the carbons and protons of C-11-C-13 and C-15, as well as an isolated proton signal at δ_H_ 9.16 (2H, br s), were indicative of an aminoimidazolinone moiety (Figure 2). Thus, **1a** was found to possess a C_11_ bromopyrrole–aminoimidazolinone moiety.

The formula identified for **1a** based on its NMR spectra accounted for C_11_H_12_BrN_5_O_2_, exactly half of the molecular formula. Furthermore, the methine groups at C-9 and C-10 required the attachment of additional groups at these positions. Overall, the dimerization of the bromopyrrole–imidazoline moiety through a cyclobutane group at C-9 and C-10 could easily account for the substituents missing from these positions. Thus, the planar structure of **1a** was identified as a dimeric sceptrin-type alkaloid. By utilizing the same NMR experiments, the planar structure of the other constituent, **1b**, was confirmed to be the same as **1a** (Table 1). A literature survey showed that oxysceptrin from the sponge *Agelas conifera* had the same kind of oxidation pattern as was seen in one of the imidazoles of sceptrin [14].

The nature of **1a** and **1b**, as well as the configurations at the cyclobutane and aminoimidazolinone stereocenters, were determined by 1D selective gradient ROESY experiments. First, conformers and diastereomers could be distinguished by NOE irradiation of paired protons [15]. For these compounds, the irradiations of 7-NH (δ_H_ 8.04) and H-11 (δ_H_ 4.45) of **1a** increased the signal intensities of only the protons in this compound, while those in **1b** were unaffected. The same phenomenon was also observed for **1b**; the irradiations of 7-NH (δ_H_ 8.24) and H-11 (δ_H_ 4.28) only changed the intensities of the signals of the protons in this compound (Figure S14). In addition, variable-temperature NMR experiments showed that the relative intensities of the key protons of **1a** and **1b** remained constant (Figure S15). Alternatively, the possibility of **1** as a mixture of carbonyl-enol tautomers was eradicated by the ^1^H NMR spectrum in MeOH-*d*_4_ in which signals of both H-11 and H-11′ were clearly observed (Figure S15). Thus, **1a** and **1b** must be epimers at either the cyclobutane or α-amide positions.

The relative configuration of the cyclobutane was assigned by ROESY experiments. The NOE cross-peaks of H_2_-8/H-10 and H-9/H-11 (also H_2_-8′/H-10′ and H-9′/H-11′) assigned the 9S*, 10R*, 9′S*, and 10′R* configurations for **1a**. The same ROESY cross-peaks for **1b** clearly indicated the *bis*-epimerization at the α-amido C-11 and C-11′ positions between these molecules. However, due to the absence of reliable ROESY correlations, the configurations at these positions remained unassigned. Both the epimerization and the unassigned configuration at the α-amide positions were consistent with what has been reported for oxysceptrin [14].

**Figure 3:**
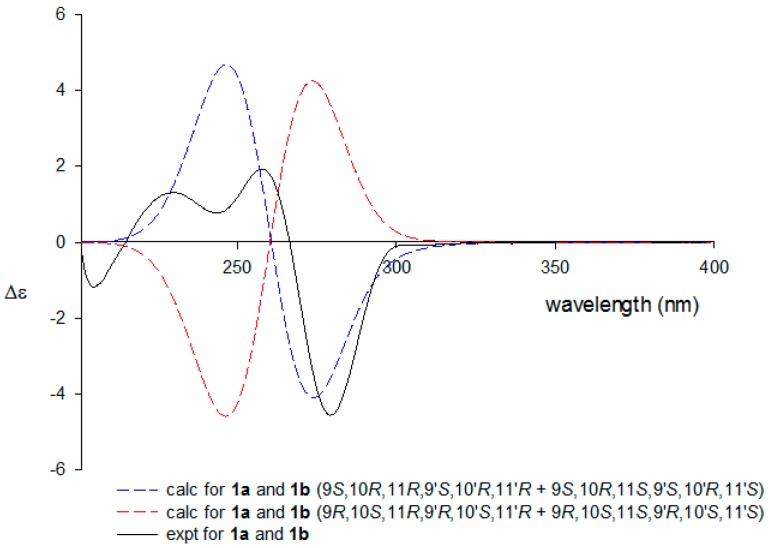
Experimental and calculated ECD spectra of **1**.

The absolute configurations of the cyclobutane core were assigned by ECD calculations. Since **1a** and **1b**, *bis*-epimers at the C-11 and C-11′ stereocenters, existed as a mixture (1:1 *v*:*v*) in **1**, both the experimental and calculated ECD data were determined in their dimeric form. The comparison of their ECD profiles clearly assigned the 9*S*, 10*R*, 9′*S*, and 10′*R* absolute configurations, which are consistent with known sceptrins (Figure 3) [9,13]. Thus, the structure of **1**, designated dioxysceptrin, was determined to be a mixture of 11,11′-dioxo derivatives of sceptrin alkaloids.

The molecular formula of ageleste C (**2**) was established to be C_18_H_18_Br_2_N_4_O_6_ (*m/z* [M + H]^+^ 544.9677, calcd. 544.9671) by HRFABMS analysis. The NMR data of this compound showed signals of nine carbons with attached protons, indicating a dimeric nature (Table 1). Comparison of the ^1^H and ^13^C NMR data with those of **1** revealed that the signals of the imidazoline moiety had been replaced with those of a carboxylic group at δ_C_ 174.0 (C-9), while those of the bromopyrrole and cyclobutane were intact (Table 1). This interpretation was confirmed by a combination of 2D NMR analyses in which the oxidative cleavage of the two imidazole moieties to carboxylic acids was clearly observed (Figure 2). Further supporting evidence was provided by comparing the spectroscopic data with those of congeners **3** and **4** in which the protons and carbons showed virtually identical chemical shifts. After the assignment of the relative configurations by ROESY experiments (Figure 2), the absolute configurations of the cyclobutane moiety were defined to be the same as those of their congeners by a comparison of their CD data (Figure S16).

In addition to **1** and **2**, eight known structurally-related bromopyrrole-bearing compounds were isolated and identified by combinations of spectroscopic methods. These compounds were ageleste A (**3**) [16], ageleste B (**4**) [16], nakamuric acid (**5**) [16,17], nakamuric acid methyl ester (**6**) [17], 4-bromopyrrole-2-carbamide (**7**) [18], 4,5-dibromopyrrole-2-carbamide (**8**) [7,19,20], dibromocantharelline (**9**) [21], and longamide B methyl ester (**10**) [22,23]. The spectroscopic data of these compounds were in good agreement with those in the literature. The isolation of **2**–**4**, bearing carboxylic acid and methyl ester groups, from this specimen suggests the possibility of methyl esterification during the separation process. The absolute configuration of **6**, which was previously unassigned, was defined as 9*S*, 10*R*, 9′*S*, and 10′*R* by comparison of its CD profile with that of nakamuric acid (**5**) (Figure S17) [16].

**Figure 4:**
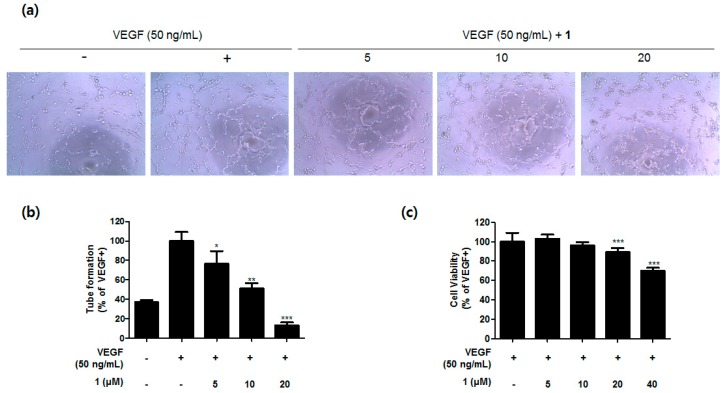
Anti-angiogenic activity of compound **1**. (**a**) Inhibitory effects of compound **1** in vascular tube formation induced by VEGF were visualized using optical microscopy. (**b**) The lengths of tubes were quantified and compared to the VEGF-treated control. (**c**) Cell viability after treatment of compound **1** in the presence of VEGF for 24 h was measured by MTT and compared to the control. Data are presented as the mean fold changes ± SD of three independent experiments. * *p* < 0.05, ** *p* < 0.01, *** *p* < 0.005 by *t*-test.

Sceptrins and structurally-related alkaloids are known to exhibit a broad range of bioactivities, such as anticancer, antibacterial, antifungal, anti-inflammatory, and anti-biofilm activities [1,3,4]. In our measurement of cytotoxicity, sceptrins were incubated with cancer cells for 72 h to assess the anti-proliferative activity. Compound **1** exhibited the most potent anti-proliferative effects against six cancer cell lines (Table 2). For anti-angiogenic activity, compounds **1**–**4** showed no cytotoxicity in HUVEC cells when treated up to 40 μM for 24 h. Subsequently, in the tube formation assay using the non-cytotoxic concentrations range (5–20 μM), only **1** exhibited moderate anti-angiogenic activity comparable to sunitinib, a positive control. The anti-angiogenic activity of compound **1** was visualized and quantified (Figure 4a–c). In antimicrobial bioassay, all the compounds were inactive (MIC > 128 μM) against a variety of human pathogenic bacterial and fungal strains. In a subsequent bioassay, contrarily, compound **2** displayed moderate inhibition of *Candida albicans*-derived isocitrate lyase (ICL), a key enzyme in microbial metabolism.

## 3. Materials and Methods

### 3.1. General Experimental Procedures

Optical rotations were measured using a JASCO P-1020 polarimeter (Easton, MD, USA) with a 1 cm cell. CD spectra were obtained using an Applied Photophysics Chirascan Plus spectrometer (Applied Photophysics Ltd., Leatherhead, Surrey, UK). UV spectra were acquired using a Hitachi U-3010 spectrophotometer (Tokyo, Japan). IR spectra were recorded on a JASCO 4200 FT-IR spectrometer (Easton, MD, USA) using a ZnSe cell. NMR spectra were recorded in DMSO-d_6_, with the solvent peaks (δ_H_ 2.50/δ_c_ 39.50) as internal standards, on a Bruker Avance 600 MHz spectrometer (Billerica, MA, USA). High-resolution FABMS spectrometric data were obtained at the National Center for Inter-university Research Facilities (NCIRF), Seoul National University and acquired using a JEOL JMS 700 mass spectrometer with 6 keV-energy, emission current 5.0 mA, xenon as inert gas, and meta-nitrobenzyl alcohol (NBA) as the matrix. HPLC separations were performed on a SpectraSYSTEM p2000 equipped with a refractive index detector (SpectraSYSTEM RI-150 (Waltham, MA, USA)) and a UV-Vis detector (Gilson UV-Vis-151 (Middleton, WI, USA)). All solvents used were of spectroscopic grade or were distilled prior to use.

### 3.2. Animal Material

Specimens of the Agelas kosrae sponge (Demospongiae: Agelasida: Agelasidae) were collected by hand using SCUBA offshore of Kosrae Island in the Federated States of Micronesia at a depth of 15 m on 23 October 2013. The sponge had an elongated repent form with several branches and had dimensions of 6 cm wide and up to 20 cm long. The texture was firm and compressible, and the color was purple on the surface and beige in the choanosome. The skeleton was composed of spicules cored primary fibres, echinated secondary fibres, and very rare achinated tertiary fibres with diameters of 100–200, 30–60, and 10–20 μm, respectively. The spicules, acanthostyles, (110–140 × 6–8 μm) and acanthoxeas (150–170 × 6–8 μm) were identical to those in the literature [24]. A voucher specimen (registry No. spo. 80) was deposited at the Natural History Museum, Hannam University, Korea, under the curatorship of C.J.S.

### 3.3. Extraction and Isolation

Freshly collected specimens were immediately frozen and stored at −25 °C until use. Lyophilized specimens (121.4 g) were macerated and repeatedly extracted with MeOH (3 × 2 L) and CH_2_Cl_2_ (3 × 2 L). The combined extracts (31.5 g) were successively partitioned between H_2_O (2.6 g) and n-BuOH (28.6 g); the latter layer was repartitioned between H_2_O-MeOH (15:85, 7.1 g) and n-hexane (21.3 g). Then, the H_2_O-MeOH layer was separated by C_18_ reversed-phase flash chromatography using sequential mixtures of MeOH and H_2_O as the eluents (six fractions in an H_2_O-MeOH gradient from 50:50 to 0:100) followed by acetone and finally EtOAc.

Based on the results of the ^1^H NMR spectroscopy and cytotoxicity analyses, the fractions that eluted with 40:60 H_2_O-MeOH (0.84 g) and 30:70 H_2_O-MeOH (1.33 g) were chosen for further separation. The 40:60 H_2_O-MeOH fraction was separated by semipreparative reversed-phase HPLC (YMC-ODS column, 10 × 250 mm; 2.0 mL/min; 20 mg/300 μL per injection; 25 °C; H_2_O-MeOH, 55:45 with 0.1% TFA, isocratic), yielding, in order of elution, compounds **9** (t_R_ = 30.7 min) and **7** (t_R_ = 40.2 min). These compounds were further purified by analytical HPLC (YMC-ODS column, 4.6 × 250 mm; 0.7 mL/min; 2 mg/200 μL per injection; 25 °C; H_2_O-MeCN gradient from 90:10 to 40:60 with 0.1% TFA in 40 min; t_R_ = 19.2 and 24.0 min, respectively).

The 30:70 H_2_O-MeOH fraction was separated by semipreparative reversed-phase HPLC (YMC-ODS column, 10 × 250 mm; 2.0 mL/min; 25 mg/300 μL per injection; 25 °C; H_2_O-MeOH, 45:55 with 0.1% TFA, isocratic), yielding, in order of elution, compounds **1**, **5**, **6**, **8**, **10**, **2**, **3**, and **4**; t_R_ = 16.5, 24.2, 28.0, 28.8, 38.5, 42.5, 48.7, and 49.1 min, respectively. Compounds **1**, **5**, **6**, **8**, and **2** were further purified by analytical HPLC under isocratic conditions (YMC-ODS column, 4.6 × 250 mm; 0.7 mL/min; 2 mg/200 μL per injection; 25 °C; H_2_O-MeCN 75:25 with 0.1% TFA, isocratic; t_R_ = 20.2, 26.0, 31.9, 28.8, and 70.1 min, respectively). Compounds **10**, **3** and **4** were also purified by analytical HPLC (H_2_O-MeCN gradient; from 75:25 to 30:70 with 0.1% TFA in 40 min); t_R_ = 14.4, 24.4, and 26.9 min, respectively. The purified metabolites were isolated in the following amounts: 13.2, 4.2, 8.2, 7.6, 3.3, 2.5, 11.6, 9.1, 7.3, and 2.7 mg of **1**–**10**, respectively.

Dioxysceptrin (**1**): yellow oil; ${\left[\alpha \right]}_{D}^{25}$ +2.9 (c 0.10, MeOH); UV (MeOH) λ_max_ (log ε) 270 (3.09) nm; CD (0.76 mM, MeOH) Δε 205 (−1.40), 230 (+1.09), 244 (+0.55), 258 (+1.71), 279 (−4.78) nm; IR (ZnSe) ν_max_ 3251, 1680, 1635, 1574, 1522, 1337 cm^−1^; ^1^H and ^13^C NMR data, Table 1; HRFABMS *m/z* 651.0432 [M + H]^+^ (calcd. for C_22_H_25_Br_2_N_10_O_4_, 651.0427).

Ageleste C (**2**): yellow oil; ${\left[\alpha \right]}_{D}^{25}$ −9.1 (c 0.12, MeOH); UV (MeOH) λ_max_ (log ε) 269 (3.34) nm; CD (0.90 mM, MeOH) Δε 211 (+0.95), 233 (−0.99), 256 (+0.39), 279 (−3.57) nm; IR (ZnSe) ν_max_ 3248, 1725, 1709, 1652, 1581, 1521 cm^−1^; ^1^H and ^13^C NMR data, Table 1; HRFABMS *m/z* 544.9677 [M + H]^+^ (calcd. for C_18_H_19_Br_2_N_4_O_6_, 544.9671).

(9S, 10R, 9′S, 10′R)-Nakamuric acid methyl ester (**6**): yellow oil; ${\left[\alpha \right]}_{D}^{25}$ −14.8 (c 0.1, MeOH); UV (MeOH) λ_max_ (log ε) 270 (3.15); CD (0.84 mM, MeOH) Δε 209 (+1.19), 230 (−1.84), 258 (+0.80), 280 (−1.68) nm.

### 3.4. ECD Calcualtions

All conformational searches were performed using Macromodel (Version 9.9, Schrodinger LLC. (New York, NY, USA)) software with “Mixed torsional/Low Mode sampling” in the MMFF force field. The searches were conducted in the gas phase with a 50 kJ/mol energy window limit and a maximum of 10,000 steps to thoroughly examine all low-energy conformers. The Polak-Ribiere conjugate gradient (PRCG) method was utilized for minimization processes with 10,000 maximum iterations and a 0.001 kJ (mol Å)^−1^ convergence threshold on the RMS gradient. Conformers within 10 kJ/mol of each global minimum for compounds **1a** and **1b** were used for gauge-independent atomic orbital (GIAO) shielding constant calculations without geometry optimization employing TmoleX Version 4.2.1 (COSMOlogic GmbH & Co. KG (Leverkusen, Germany)) at the B3LYP/6-31G(d,p) level in the gas phase. The CD spectra were simulated by overlapping each transition, where σ is the width of the band at 1/e height. ΔE_i_ and R_i_ are the excitation energies and rotatory strengths, respectively, for transition i. In the current work, the value of σ was 0.10 eV.

$$\Delta \in \left(E\right)=\frac{1}{2.297\times 10^{-39}}\frac{1}{\surd 2\pi \sigma }\overset{A}{\underset{i}{\sum }}{\Delta E}_{i}R_{i}e^{\left[-{\left(E-\Delta E_{i}\right)}^{2}/{\left(2\sigma \right)}^{2}\right]}$$

### 3.5. Anti-Proliferative Activity Assay

Anti-proliferative activity was evaluated using SRB staining assay in various cancer cell lines (K562, A549, HCT116, MDA-MB-231, SNU638, SK-Hep-1). Cells were purchased from the American Type Culture Collection (ATCC, Rockville, MD, USA). They were cultured in media supplemented with 10% fetal bovine serum (FBS) and antibiotics–antimycotics (PSF; 100 units/mL penicillin G sodium, 100 ng/mL streptomycin, and 250 ng/mL amphotericin B). All cells were maintained at 37 °C under a humidified atmosphere containing 5% CO_2_. Briefly, cells were seeded in 96-well plates with various doses of compounds and incubated for 72 h. The cells were stained as previously described [25]. First, cells were fixed with 10% trichloroacetic acid and stained with 0.4% SRB in a 1% acetic acid solution. After washing and drying, dyes were dissolved in 10 mM Tris buffer (pH 10.0) and absorbance was measured at 515 nm. The percentage of cell proliferation was determined according to the following formula: cell proliferation (%) = 100 × ((A treated − A zero day)/(A control − A zero day)), where A is the average absorbance. The IC_50_ values were calculated through non-linear regression analysis using TableCurve 2D v5.01 (Systat Software Inc., San Jose, CA, USA).

### 3.6. Anti-Angiogenic Activity Assay

Anti-angiogenic activity was evaluated using human umbilical vein endothelial cells (HUVEC) using previously described experimental methods [26]. HUVEC cells were purchased from the American Type Culture Collection (ATCC, Rockville, MD, USA) and cultured in EGM-2 (Lonza, Walkerswille, MD, USA) supplemented with 10% fetal bovine serum (FBS) and antibiotics–antimycotics (PSF; 100 units/mL penicillin G sodium, 100 ng/mL streptomycin, and 250 ng/mL amphotericin B). Briefly, HUVEC cells were mixed with tested compounds in 0.5% FBS EBM-2 media stimulated with or without VEGF (50 ng/mL) on matrigel-coated 96-well plates for 6 h at 37 °C under a humidified atmosphere containing 5% CO_2_. After the tube formation, cells were photographed using an inverted microscope (Olympus Optical Co. Ltd., Tokyo, Japan), then images were quantified with Angiogenesis Analyzer using Image J software. Tube formation activity was calculated using the following formula: (Total segment # (tested compound) − Total segment # (VEGF−))/(Total segment # (VEGF+) − Total segment # (VEGF−)) × 100. The IC_50_ value was calculated through non-linear regression analysis using TableCurve 2D v5.01 (Systat Software Inc., San Jose, CA, USA). Cell viability with HUVEC cells were measured independently. First, HUVEC cells were seeded into a 96-well plate and the culture medium was replaced with a serum-free medium when it reached 60% confluency. After overnight starvation, cells were treated with samples and VEGF (50 ng/mL) in 2% FBS EBM-2 medium. Cells were further incubated for 24 h, and MTT assay was used to measure the cell viability. The formazan products were dissolved in dimethyl sulfoxide (DMSO). The absorbance was measured at 570 nm using VersaMax ELISA microplate reader (Molecular Devices, Sunnyvale, CA, USA).

### 3.7. Isocitrate lyase (ICL) Activity Assay

A 1 mL aliquot of the reaction mixture contained 20 mM sodium phosphate buffer (pH 7.0), 1.27 mM threo-DL-(+)-isocitrate, 3.75 mM MgCl_2_, 4.1 mM phenylhydrazine, and 2.5 μg/mL of recombinant ICL. The reaction was immediately initiated following the addition of a substrate with or without a prescribed concentration of the inhibitor dissolved in DMSO (final concentration, 1%). Glyoxylate phenylhydrazone formation was spectrophotometrically assessed at 324 nm after incubation at 37 °C for 30 min. The percent inhibition of ICL enzyme activity for each compound was calculated relative to the inhibitor-free control and the IC_50_ values were calculated using nonlinear regression analysis (percent inhibition versus concentration). 3-Nitropropionic acid was used as a positive control. Protein concentrations were measured using the Bradford method with the Bio-Rad protein assay kit (Bio-Rad) and bovine serum albumin as the standard.

### 3.8. Antibacterial Activity Assay

Gram-positive bacteria (*Staphylococcus aureus* ATCC 25923, *Enterococcus faecalis* ATCC 19433 and *Enterococcus faecium* ATCC 19434) and Gram-negative bacteria (*Klebsiella pneumoniae* ATCC 10031, *Salmonella enterica* ATCC 14028 and *Escherichia coli* ATCC 25922) were used for antibacterial activity tests. Bacteria were grown overnight in Mueller Hinton (MH) broth at 37 °C, harvested by centrifugation and washed twice with sterile distilled water. Stock solutions of the compound were prepared in DMSO. Each stock solution was diluted with MH broth to give serial two-fold dilutions in the range of 128 to 0.06 μg/mL. The final DMSO concentration was maintained at 1% by adding DMSO to the MB broth. Aliquots (10 μL) of the broth containing approximately 5 × 10^5^ colony-forming units (cfu)/mL of the bacteria were added to each well of a 96-well plate. The plates were incubated for 24 h at 37 °C. The minimum inhibitory concentration (MIC) values were determined as the lowest concentration of the test compound that inhibited bacterial growth. Ampicillin and tetracycline were used as reference compounds.

### 3.9. Antifungal Activity Assay

Potato dextrose agar (PDA) was used to cultivate *Candida albicans* ATCC 10231. After incubation for 48 h at 28 °C, yeast cells were harvested by centrifugation and washed twice with sterile distilled water. *Aspergillus fumigatus* HIC 6094, *Trichophyton rubrum* NBRC 9185 and *Trichophyton mentagrophytes* IFM 40996 were plated on PDA and incubated for 2 weeks at 28 °C. Spores were harvested and washed twice with sterile distilled water. Stock solutions of the compound were prepared in DMSO. Each stock solution was diluted with RPMI 1640 broth (Difco) to give serial two-fold dilutions in the range of 128 to 0.06 μg/mL. The final DMSO concentration was maintained at 1% by adding DMSO to the broth. Aliquots (10 μL) of the RPMI 1640 broth containing approximately 10^4^ cells/mL were mixed with the test compound solutions in each well of a 96-well plate. The plates were incubated for 24 h (for *C. albicans*), 48 h (for *A. fumigatus*) and 96 h (for *T. rubrum* and *T. mentagrophytes*) at 37 °C. A culture with DMSO (1%) was used as a solvent control, and a culture supplemented with amphotericin B was used as a positive control.

### 3.10. Disk Diffusion Assay

Gram-positive bacteria (*S. aureus* ATCC 25923 and *Bacillus subtilis* ATCC 6633) were used for disk diffusion assay. Bacteria were grown overnight in Mueller Hinton (MH) broth at 37 °C, harvested by centrifugation and washed twice with sterile distilled water. Stock solutions of the compound were prepared in DMSO. Bacterial cells (5 × 10^5^ colony-forming units (cfu)/mL) were inoculated and 128 μg/disk of the compound was added to each agar plate. The plates were incubated for 24 h at 37 °C. Ampicillin was used as a reference compound.

## 4. Conclusions

Two new sceptrin derivatives (**1**,**2**) and eight structurally-related known bromopyrrole-bearing alkaloids were isolated from the tropical sponge *Agelas kosrae.* The structure elucidation of compounds **1** and **2** were established by combined spectroscopic methods. Dioxysceptrin (**1**) was also found to exist as a mixture of α-amido epimers. Absolute configurations were determined by comparison of the experimental and calculated ECD data. The sceptrin alkaloids exhibited weak cytotoxicity against cancer cell-lines. Compounds **1** and **2** also moderately exhibited anti-angiogenic and isocitrate lyase-inhibitory activities, respectively.

## Figures and Tables

**Table 1:** NMR spectral data for compounds **1** and **2** in DMSO-*d*_6_ (600 MHz).

Position	1a		1b		2	
	δ_H_, mult (*J* in Hz)	δ_C_ (Type)	δ_H_, mult (*J* in Hz)	δ_C_ (Type)	δ_H_, mult (*J* in Hz)	δ_C_ (Type)
1/1′	11.73, br s		11.79, br s		11.80, br s	
2/2′	6.95, m	121.3 (CH)	6.98, m	121.3 (CH)	6.97, dd (2.7, 1.5)	121.2 (CH)
3/3′		95.1 (C)		95.0 (C)		94.9 (C)
4/4′	6.84, m	111.8 (CH)	6.85, m	111.6 (CH)	6.84, dd (2.7, 1.5)	111.5 (CH)
5/5′		126.6 (C)		126.7 (C)		126.7 (C)
6/6′		160.2 (C)		160.0 (C)		160.0 (C)
7/7′	8.04, t (5.7)		8.24, t (5.7)		8.19, t (5.8)	
8/8′	3.26, m; 3.13, m	41.9 (CH_2_)	3.33, m; 3.27, m	41.6 (CH_2_)	3.36, m; 3.25, m	40.8 (CH_2_)
9/9′	2.08, m	37.1 (CH)	2.07, m	38.2 (CH)	2.25, m	38.9 (CH)
10/10′	2.47, m	38.2 (CH)	2.46, m	36.7 (CH)	2.83, m	40.5 (CH)
11/11′	4.45, s	60.3 (CH)	4.28, s	60.0 (CH)		174.0 (C)
12/12′	10.01, s		10.07, s			
13/13′		158.7 (C)		158.7 (C)		
14/14′						
15/15′		174.3 (C)		174.1 (C)		
16/16′	9.16, br s		9.16, br s			

**Table 2:** Results of bioactivity test.

	IC_50_ (μM)									
		Anti-Proliferation						Anti-Angiogenesis		
Compound	K562	A549	HCT 116	MDA-MB-231	SNU 638	SK-Hep-1		Tube Formation	Cell Viability	ICL
**1**	12.87	31.32	9.93	32.83	7.92	35.19		7.99	>40	>100
**2**	19.13	49.32	44.06	>50	36.77	>50		>40	>40	22.09
**3**	37.61	>50	>50	>50	>50	>50		>40	>40	>100
**4**	26.42	30.5	38.19	>50	37.06	>50		>40	>40	85.31
Doxorubicin	1.08									
Etoposide		0.83	0.73	3.12	0.63	0.74				
Sunitinib								2.41	9.92	
3-NP										11.09

## Supplementary Materials

The following are available online at http://www.mdpi.com/1660-3397/16/12/513/s1, Figure S1–S12: annotated 1D NMR and selected 2D NMR spectra of **1** and **2**, Figure S13: ESI/MS isotopic cluster patterns of **1, 2** and **6**, Figure S14: 1D Selective gradient ROESY experiments of **1**, Figure S15: The variable temperature NMR experiments of **1**, Figure S16-S17: The CD spectrum of **2**–**4** and **6**.

## References

[1] Rane R., Sahu N., Shah C., Karpoormath R. (2014). Marine bromopyrrole alkaloids: Synthesis and diverse medicinal applications. Curr. Top. Med. Chem..

[2] Ancheeva E., El-Neketi M., Song W., Lin W., Daletos G., Ebrahim W., Proksch P. (2017). Structurally unprecedented metabolites from marine sponges. Curr. Org. Chem..

[3] Lindel T. (2017). Chemistry and biology of the pyrrole-imidazole alkaloids. Alkaloids.

[4] Zhang H., Dong M., Chen J., Wang H., Tenney K., Crews P. (2017). Bioactive secondary metabolites from the marine sponge genus *Agelas*. Mar. Drugs.

[5] Burkholder P.R., Sharma G.M. (1969). Antimicrobial agents from the sea. Lloydia.

[6] Sharma G.M., Burkholder P.R. (1971). Structure of dibromophakellin, a new bromine-containing alkaloid from the marine sponge *Phakellia flabellate*. J. Chem. Soc. Chem. Commun..

[7] Forenza S., Minale L., Riccio R., Fattorusso E. (1971). New bromopyrrole derivatives from the sponge *Agelas oroides*. J. Chem. Soc. Chem. Commun..

[8] Garcia E.E., Benjamin L.E., Fryer R.I. (1973). Reinvestigation into the structure of oroidin, a bromopyrrole derivative from marine sponge. J. Chem. Soc. Chem. Commun..

[9] Walker R.P., Faulkner D.J., Van Engen D., Clardy J. (1981). Sceptrin, an antimicrobial agent from the sponge *Agelas sceptrum*. J. Am. Chem. Soc..

[10] Wang X., Ma Z., Wang X., De S., Ma Y., Chen C. (2014). Dimeric pyrrole-imidazole alkaloids: Synthetic approaches and biosynthetic hypotheses. Chem. Commun..

[11] Beniddir M.A., Evanno L., Joseph D., Skiredj A., Poupon E. (2016). Emergence of diversity and stereochemical outcomes in the biosynthetic pathways of cyclobutane-centered marine alkaloid dimers. Nat. Prod. Rep..

[12] Forte B., Malgesini B., Piutti C., Quartieri F., Scolaro A., Papeo G. (2009). A submarine journey: The pyrrole-imidazole alkaloids. Mar. Drugs.

[13] Ma Z., Wang X., Wang X., Rodriguez R.A., Moore C.E., Gao S., Tan S., Ma Y., Rheingold A.L., Baran P.S. (2014). Asymmetric syntheses of sceptrin and massadine and evidence for biosynthetic enantiodivergence. Science.

[14] Keifer P.A., Schwartz R.E., Koker M.E.S., Hughes R.G., Rittschof D., Rinehart K.L. (1991). Bioactive bromopyrrole metabolites from the Caribbean sponge *Agelas conifera*. J. Org. Chem..

[15] Hu D.X., Grice P., Ley S.V. (2012). Rotamers or diastereomers? An overlooked NMR solution. J. Org. Chem..

[16] Sun Y.-T., Lin B., Li S.-G., Liu M., Zhou Y.-J., Xu Y., Hua H.-M., Lin H.-W. (2017). New bromopyrrole alkaloids from the marine sponge *Agelas* sp.. Tetrahedron.

[17] Eder C., Proksch P., Wray V., van Soest R.W.M., Ferdinandus E., Pattisina L.A. (1999). New bromopyrrole alkaloids from the Indopacific sponge *Agelas nakamurai*. J. Nat. Prod..

[18] Mancini I., Guella G., Amade P., Roussakis C., Pietra F. (1997). Hanishin, a semiracemic, bioactive C9 alkaloid of the axinellid sponge *Acanthella carteri* from the Hanish Islands. A shunt metabolite?. Tetrahedron Lett..

[19] Tada H., Tozko T. (1988). Two bromopyrroles from a marine sponge *Agelas* sp.. Chem. Lett..

[20] Tsukamoto S., Kate H., Hirota H., Fusetani N. (1996). Mauritiamine, a new antifouling oroidin dimer from the marine sponge *Agelas mauritiana*. J. Nat. Prod..

[21] De Nanteuil G., Ahond A., Guilhem J., Poupat C., Dau E.T.H., Potier P., Pusset M., Pusset J., Laboute P. (1985). Marine invertebrates from the new Caledonian lagoon. V. Isolation and identification of metabolites of a new species of sponge, *Pseudaxinyssa cantharella*. Tetrahedron.

[22] Umeyama A., Ito S., Yuasa E., Arihara S., Yamada T. (1998). A new bromopyrrole alkaloid and the optical resolution of the racemate from the marine sponge *Homaxinella* sp.. J. Nat. Prod..

[23] Reddy N.S., Venkateswarlu Y. (2000). S-(+)-methyl ester of hanishin from the marine sponge *Agelas ceylonica*. Biochem. Syst. Ecol..

[24] Sim C.J., Kim Y.A. (2014). Six new agelas species (*Demospongiae*: *Agelasida*: *Agelasidae*) from Kosrae Island, the Federated States of Micronesia. Anim. Syst. Evol. Divers..

[25] Kim W.K., Byun W.S., Chung H.J., Oh J., Park H.J., Choi J.S., Lee S.K. (2018). Esculetin suppresses tumor growth and metastasis by targeting Axin2/E-cadherin axis in colorectal cancer. Biochem. Pharmacol..

[26] Yu S., Oh J., Li F., Kwon Y., Cho H., Shin J., Lee S.K., Kim S. (2017). New scaffold for angiogenesis inhibitors discovered by targeted chemical transformations of wondonin natural products. ACS Med. Chem. Lett..

