# Supplementary material for: Bromopyrrole Alkaloids from the Sponge Agelas kosrae
Source: Mar Drugs. 2018 Dec 17;16(12):513. doi: 10.3390/md16120513 (PMC6316234; doi:10.3390/md16120513)
Supplement: Supplementary file 1 [file marinedrugs-16-00513-s001.pdf]

Supporting Information

Bromopyrrole alkaloids from the sponge Agelas kosrae

Oh-Seok Kwon [1], Donghwa Kim [1], Heegyu Kim [2], Yeon-Ju Lee [3], Hyi-Seung Lee [3], Chung J. Sim [4], Dong-Chan Oh [1], Sang Kook Lee [1], Ki-Bong Oh [2,*], and Jongheon Shin [1,*]

[1] Natural Products Research Institute, College of Pharmacy, Seoul National University, San 56-1, Sillim, Gwanak, Seoul 151-742, Korea; ideally225@snu.ac.kr (O.-S. K.); kdh9298@gmail.com (D. K.); dongchanoh@snu.ac.kr (D.-C. O.); sklee61@snu.ac.kr (S.K.L.); shinj@snu.ac.kr (J. S.)

[2] Department of Agricultural Biotechnology, College of Agriculture and Life Science, Seoul National University, San 56-1, Sillim, Gwanak, Seoul 151-921, Korea; hqhqeori@snu.ac.kr (H. K.); ohkibong@snu.ac.kr (K.-B. O.)

[3] Marine Natural Products Laboratory, Korea Institute of Ocean Science & Technology, P.O. Box 29, Seoul 425-600, Korea; yjlee@kiost.ac.kr (Y.-J. L.); hslee@kiost.ac.kr (H.-S. L.)

[4] Department of Biological Science, College of Life Science and Nano Technology, Hannam University, 461-6 Jeonmin, Yuseong, Daejeon 305-811, Korea; cjs@hnu.kr (C. J. S.)

* Correspondence: shinj@snu.ac.kr (J. S.); ohkibong@snu.ac.kr (K.-B. O.); Tel.: +82-2-880-2484 (J. S.); +82-2-880-4646 (K.-B. O.)

Figure S1. The ^{1}H NMR (600 MHz, DMSO-d_6) spectrum of **1** S3

Figure S2. The ^{13}C NMR (150 MHz, DMSO-d_6) spectrum of **1** S4

Figure S3. The COSY (600 MHz, DMSO-d_6) spectrum of **1** S5

Figure S3. The TOCSY (600 MHz, DMSO-d_6) spectrum of **1** S6

Figure S4. The eHSQC (600 MHz, DMSO-d_6) spectrum of **1** S7

Figure S5. The HMBC (600 MHz, DMSO-d_6) spectrum of **1** S8

Figure S6. The ROESY (600 MHz, DMSO-d_6) spectrum of **1** S9

Figure S7. The ^{1}H NMR (600 MHz, DMSO-d_6) spectrum of **2** S10

Figure S8. The ^{13}C NMR (150 MHz, DMSO-d_6) spectrum of **2** S11

Figure S9. The COSY (600 MHz, DMSO-d_6) spectrum of **2** S12

Figure S10. The eHSQC (600 MHz, DMSO-d_6) spectrum of **2** S13

Figure S11. The HMBC (600 MHz, DMSO-d_6) spectrum of **2** S14

Figure S12. The ROESY (600 MHz, DMSO-d_6) spectrum of **2** S15

Figure S13. ESI/MS isotopic cluster patterns of compounds 1, 2 and 6 ((a)-(c)) in positive and negative ion modes S16

Figure S14. 1D Selective gradient ROESY experiments (600 MHz, DMSO-d_6) of **1** S18

Figure S15. The variable temperature NMR experiments (600 MHz, MeOH-d_4 and DMSO-d_6) of **1** S21

Figure S16. The CD spectra of **2-4** S22

Figure S17. The CD spectrum of **6** S23

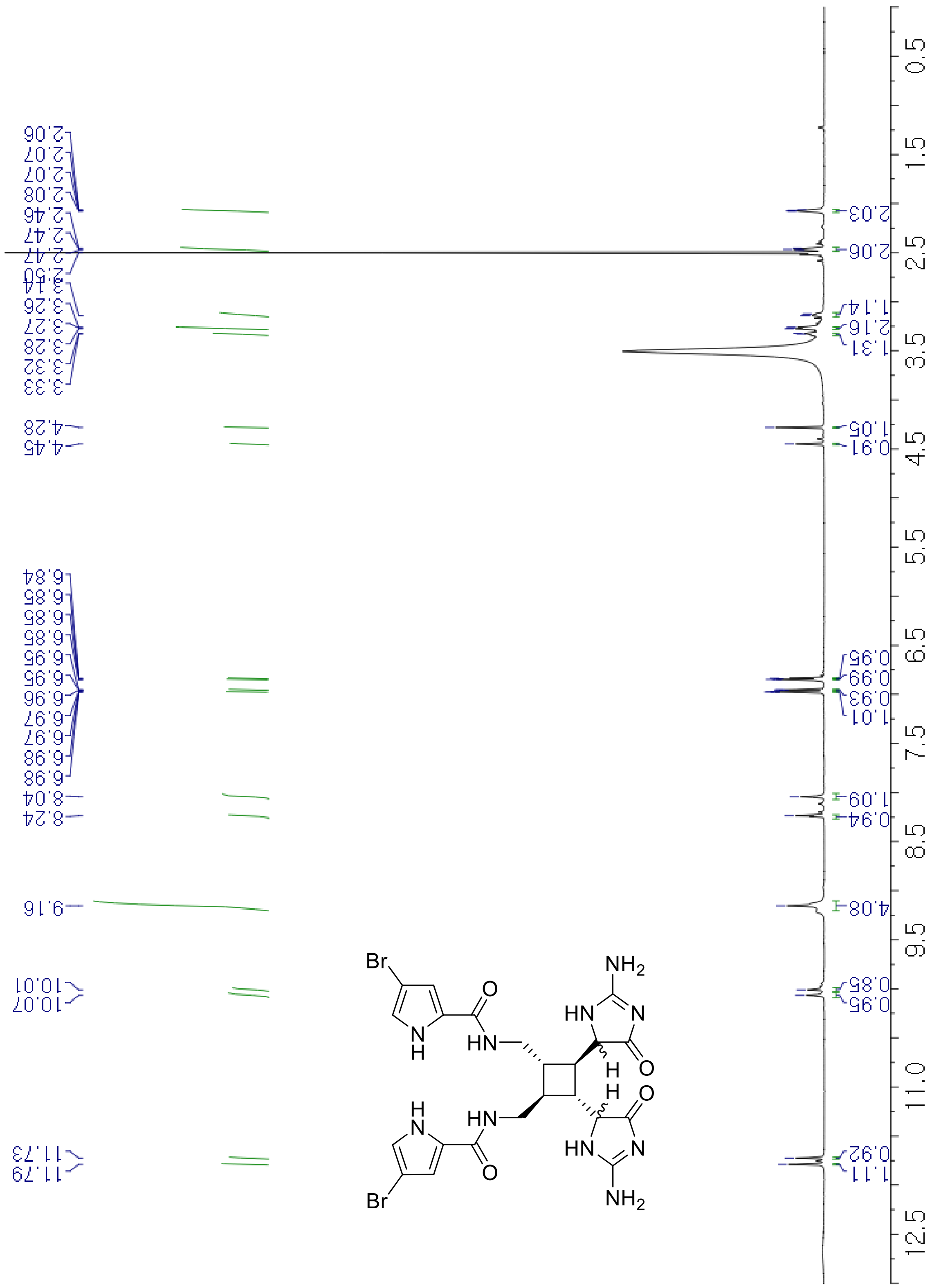

Figure S1. The ^{1}H NMR (600 MHz, DMSO-d_6) spectrum of **1**

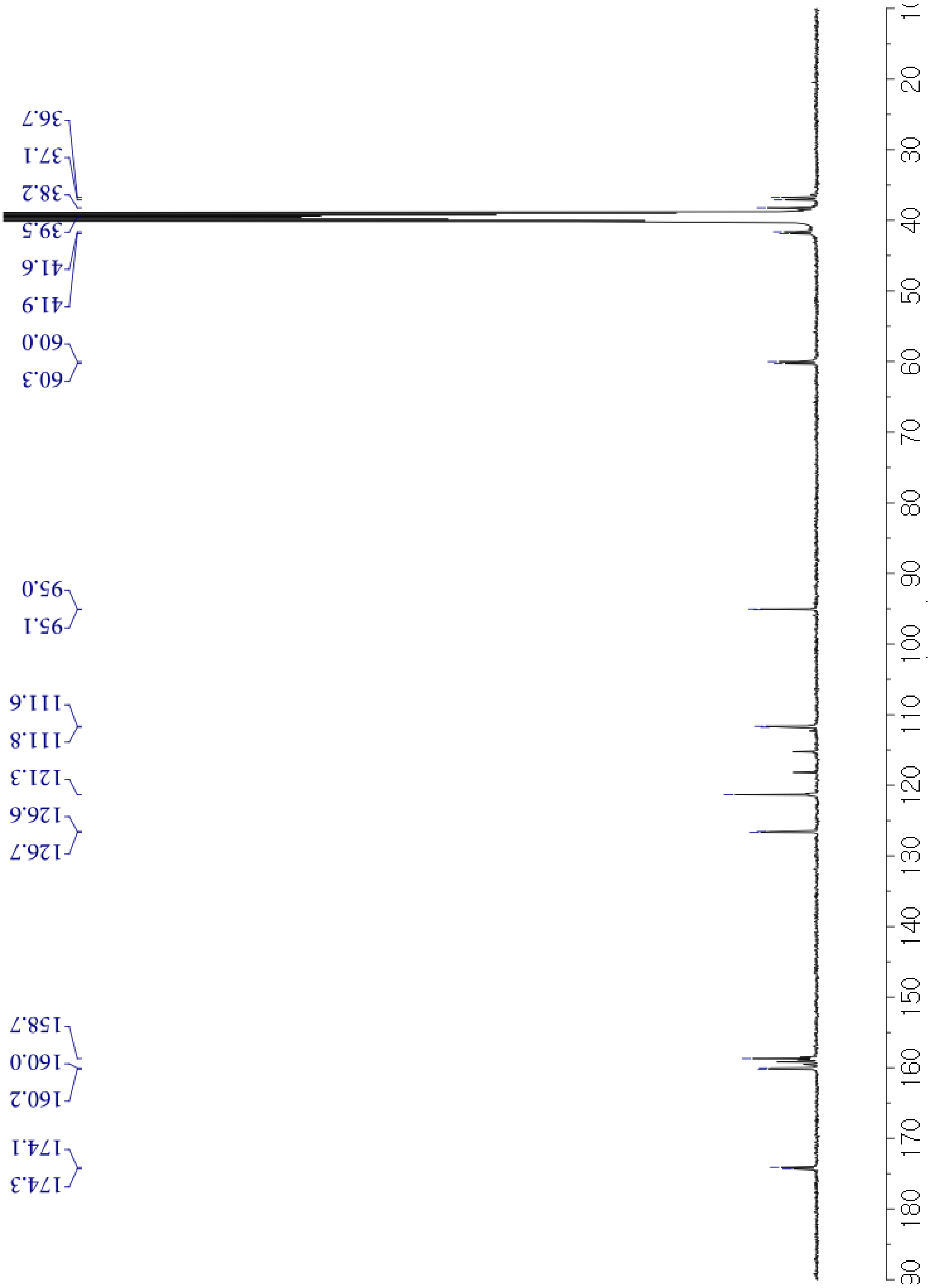

Figure S2. The ^{13}C NMR (150 MHz, DMSO-d_6) spectrum of **1**

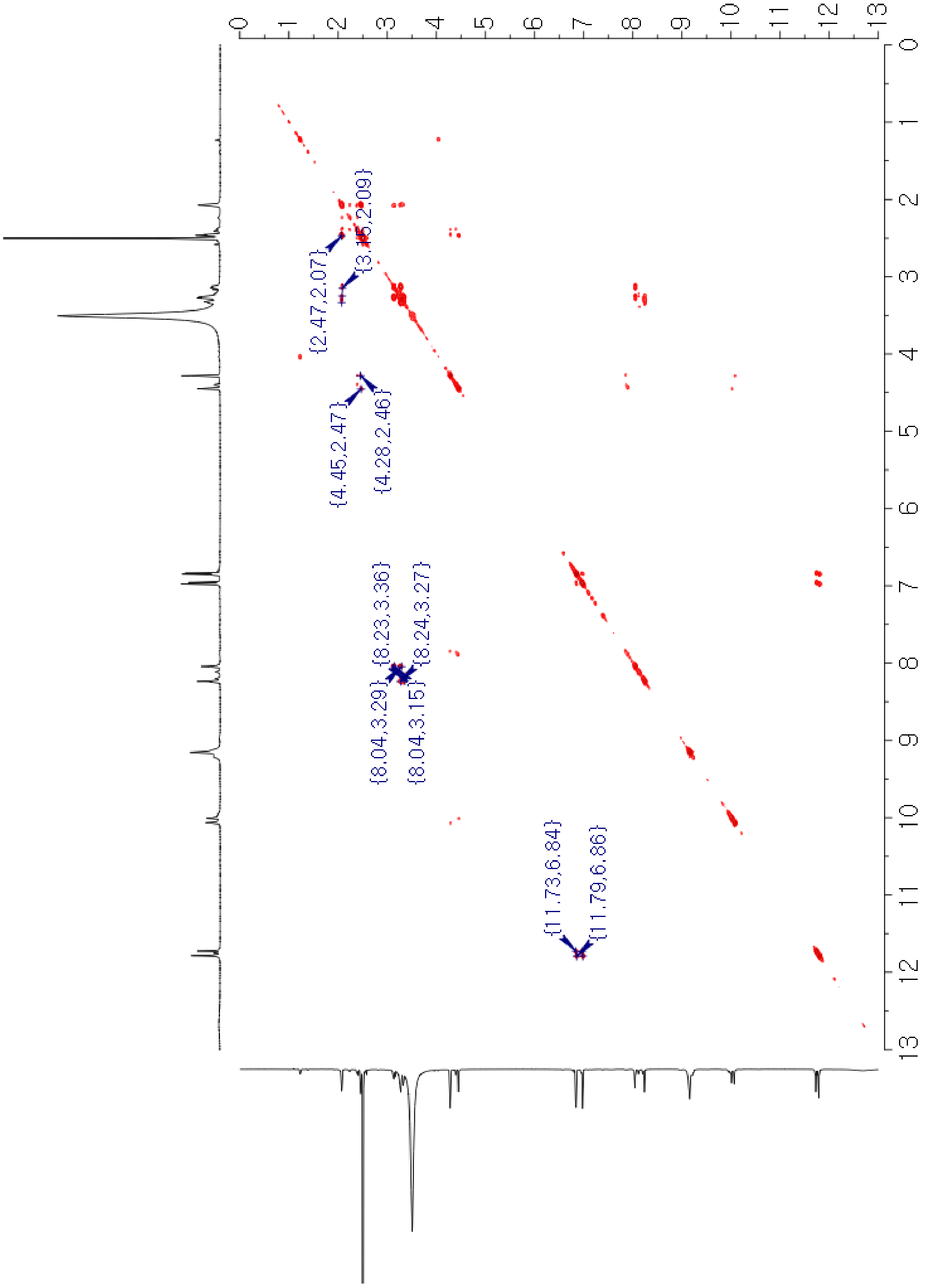

Figure S3. The COSY (600 MHz, DMSO-d_6) spectrum of **1**

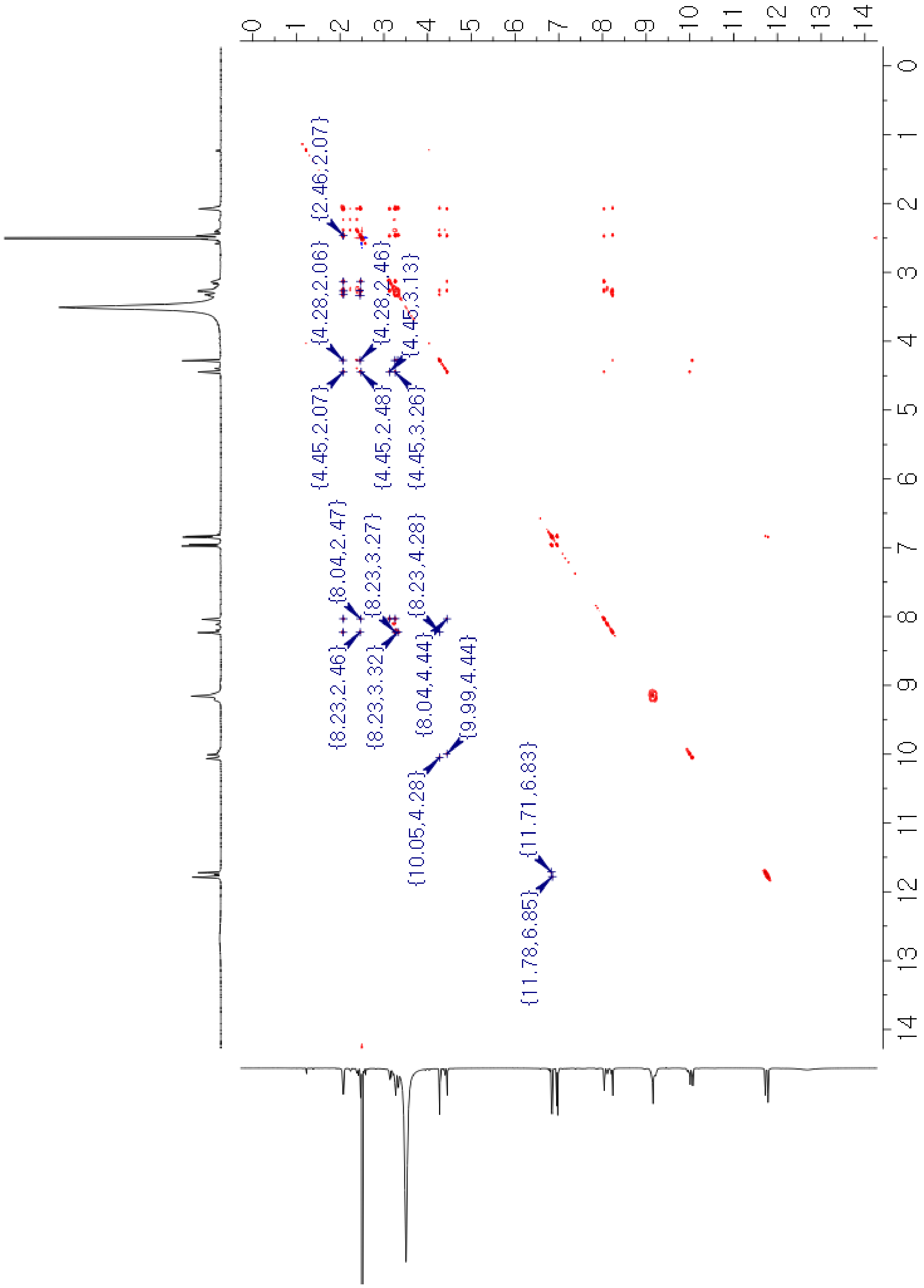

Figure S3. The TOCSY (600 MHz, DMSO-d_6) spectrum of **1**

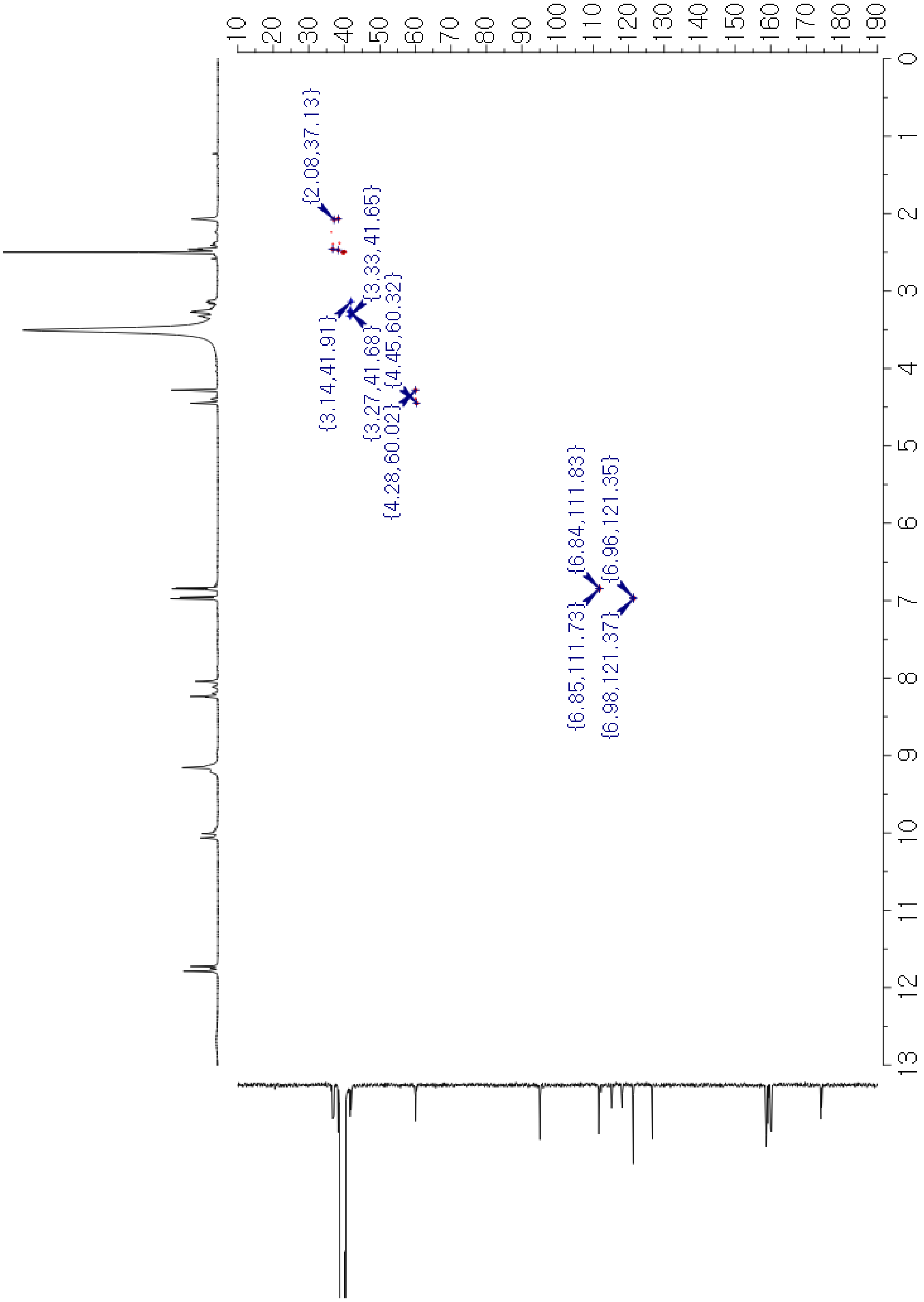

Figure S4. The eHSQC (600 MHz, DMSO-d_6) spectrum of **1**

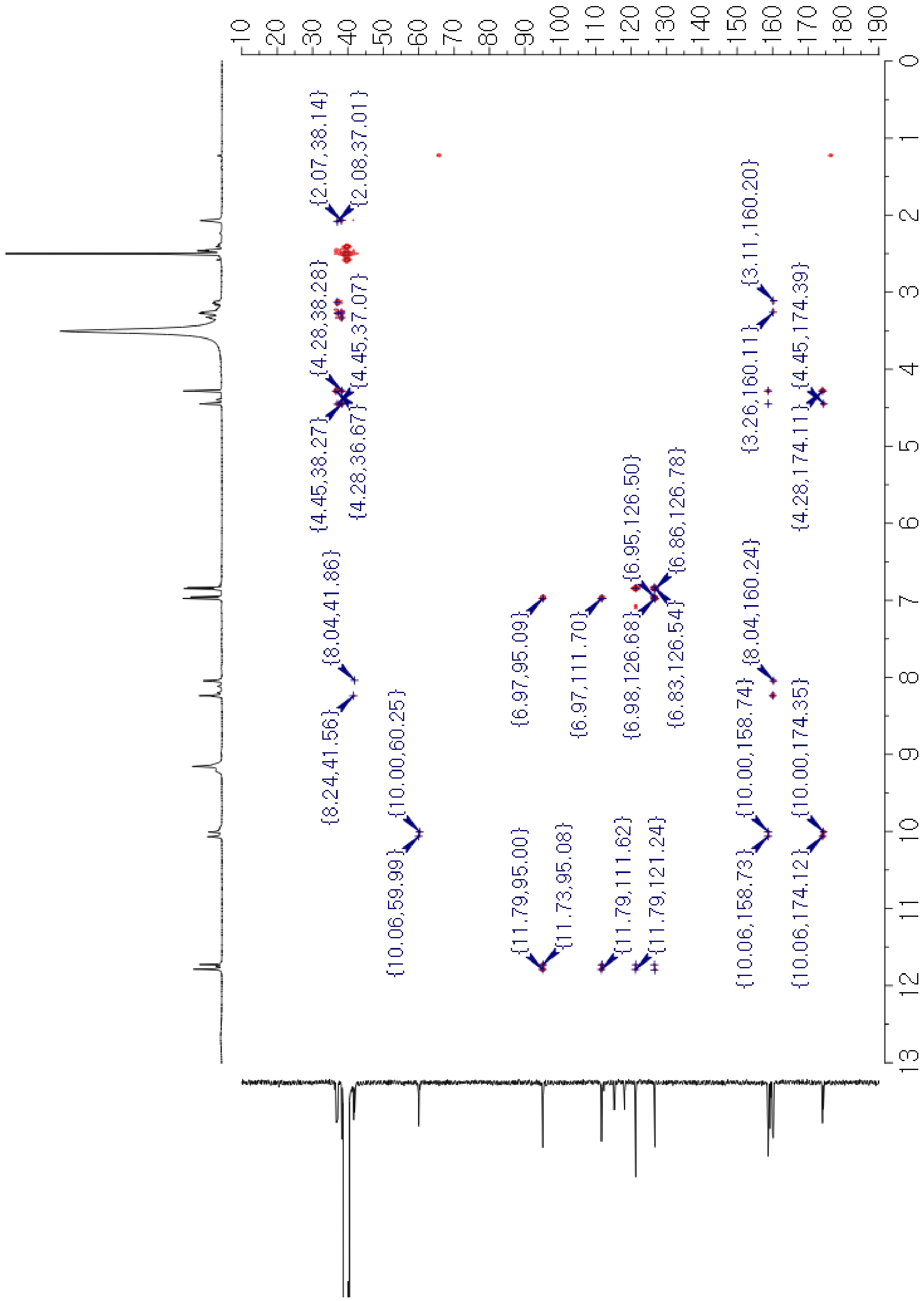

Figure S5. The HMBC (600 MHz, DMSO-d_6) spectrum of **1**

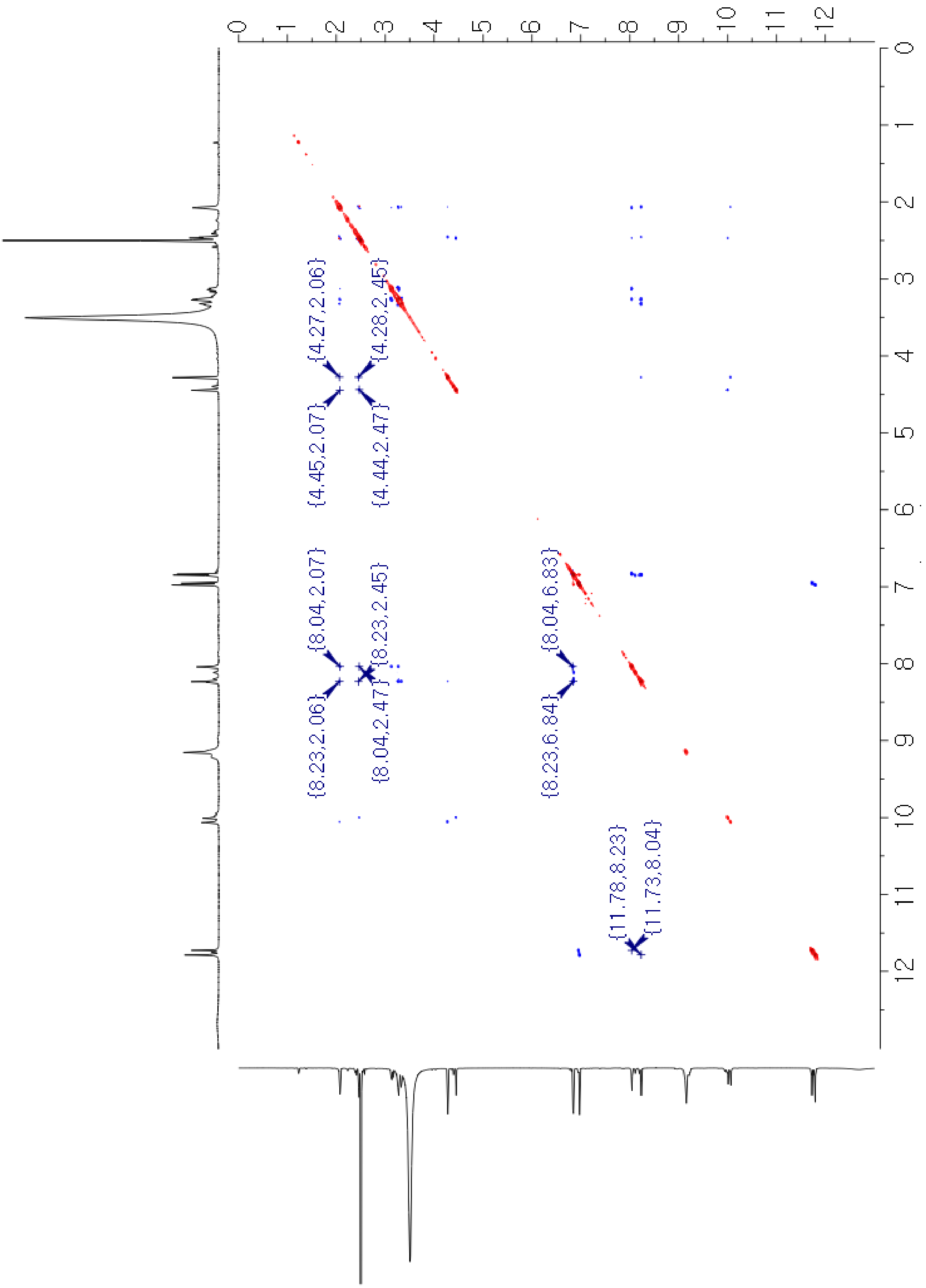

Figure S6. The ROESY (600 MHz, DMSO-d_6) spectrum of **1**

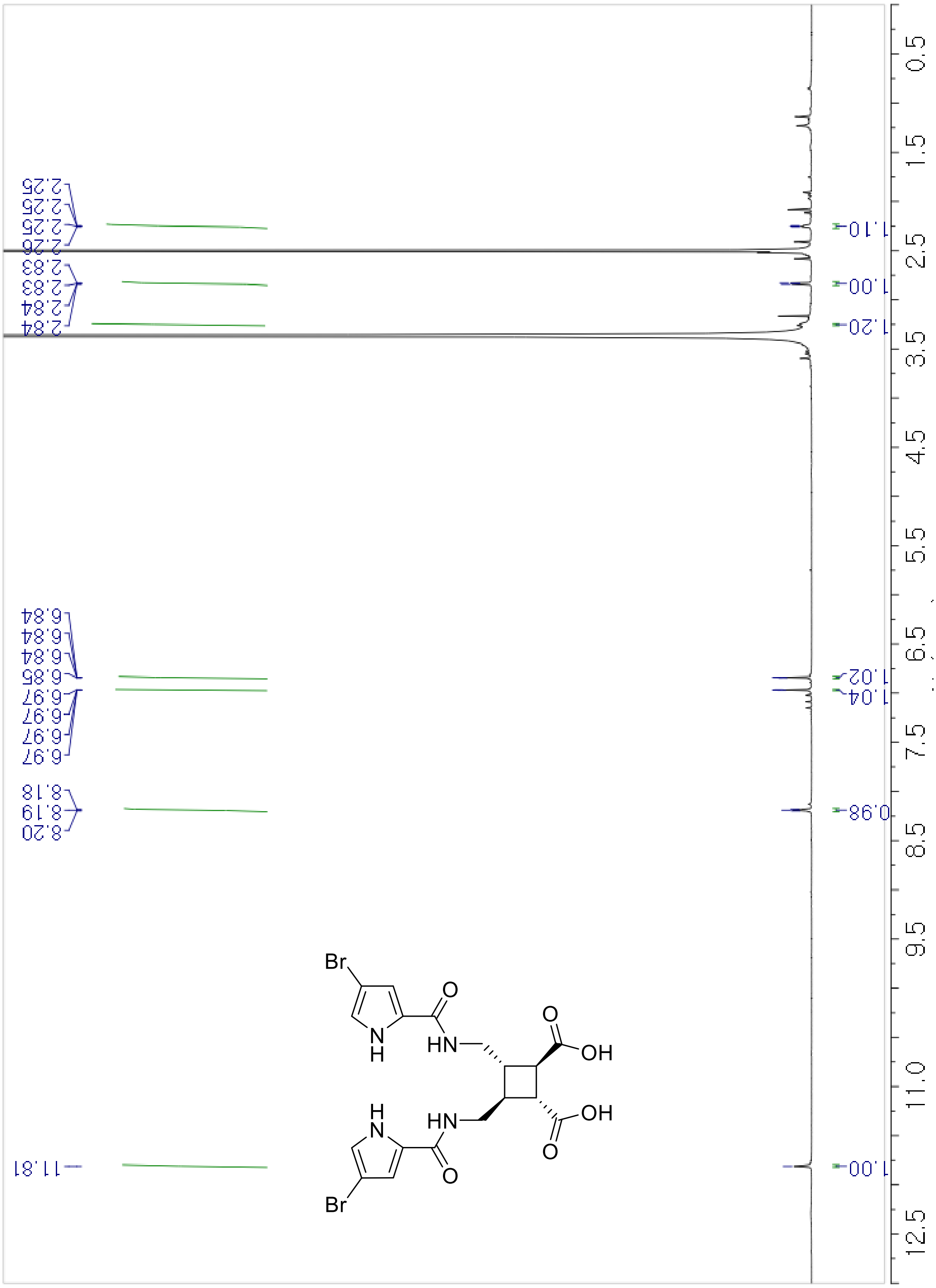

Figure S7. The ^{1}H NMR (600 MHz, DMSO-d_6) spectrum of **2**

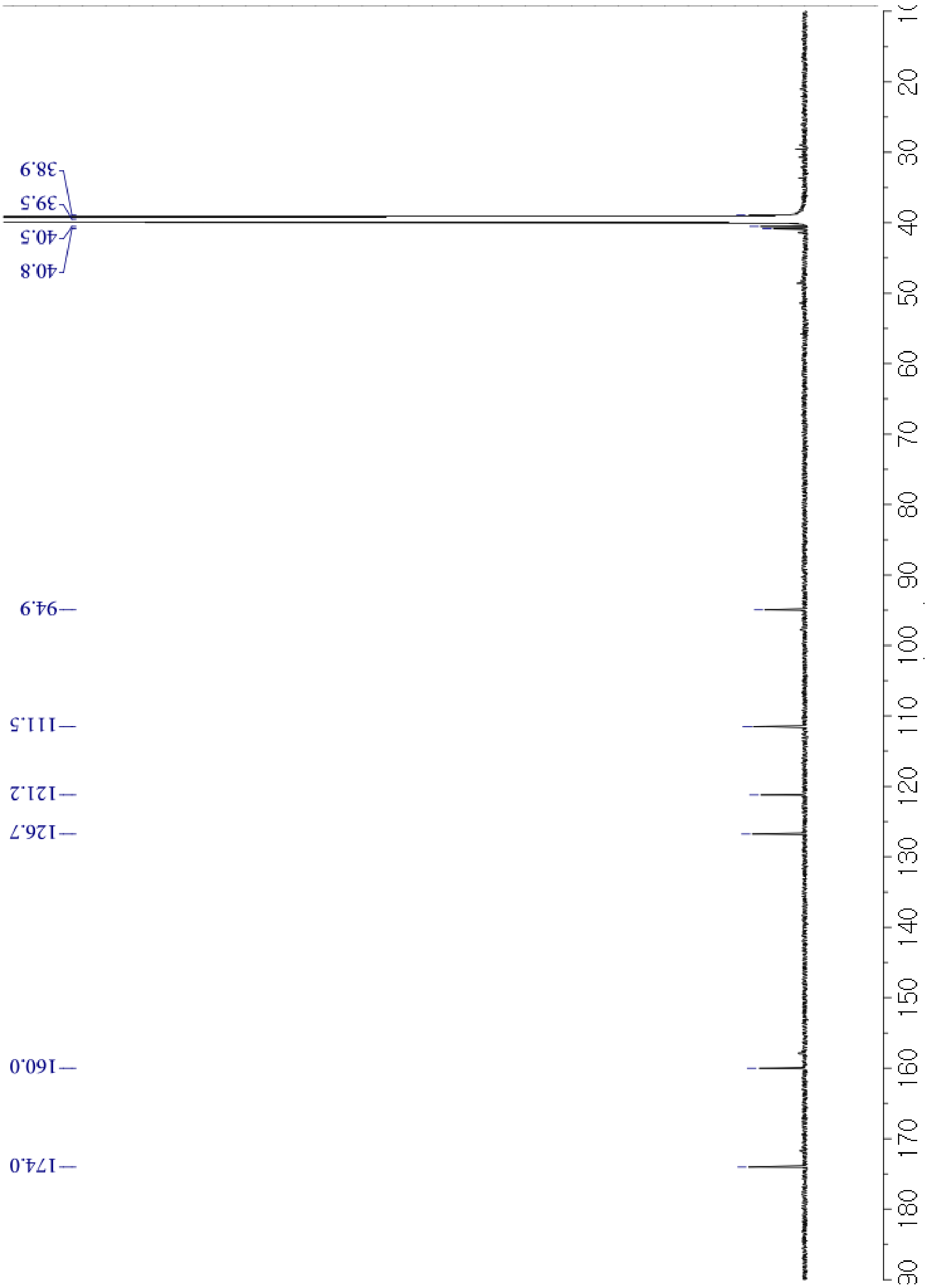

Figure S8. The ^{13}C NMR (150 MHz, DMSO-d_6) spectrum of **2**

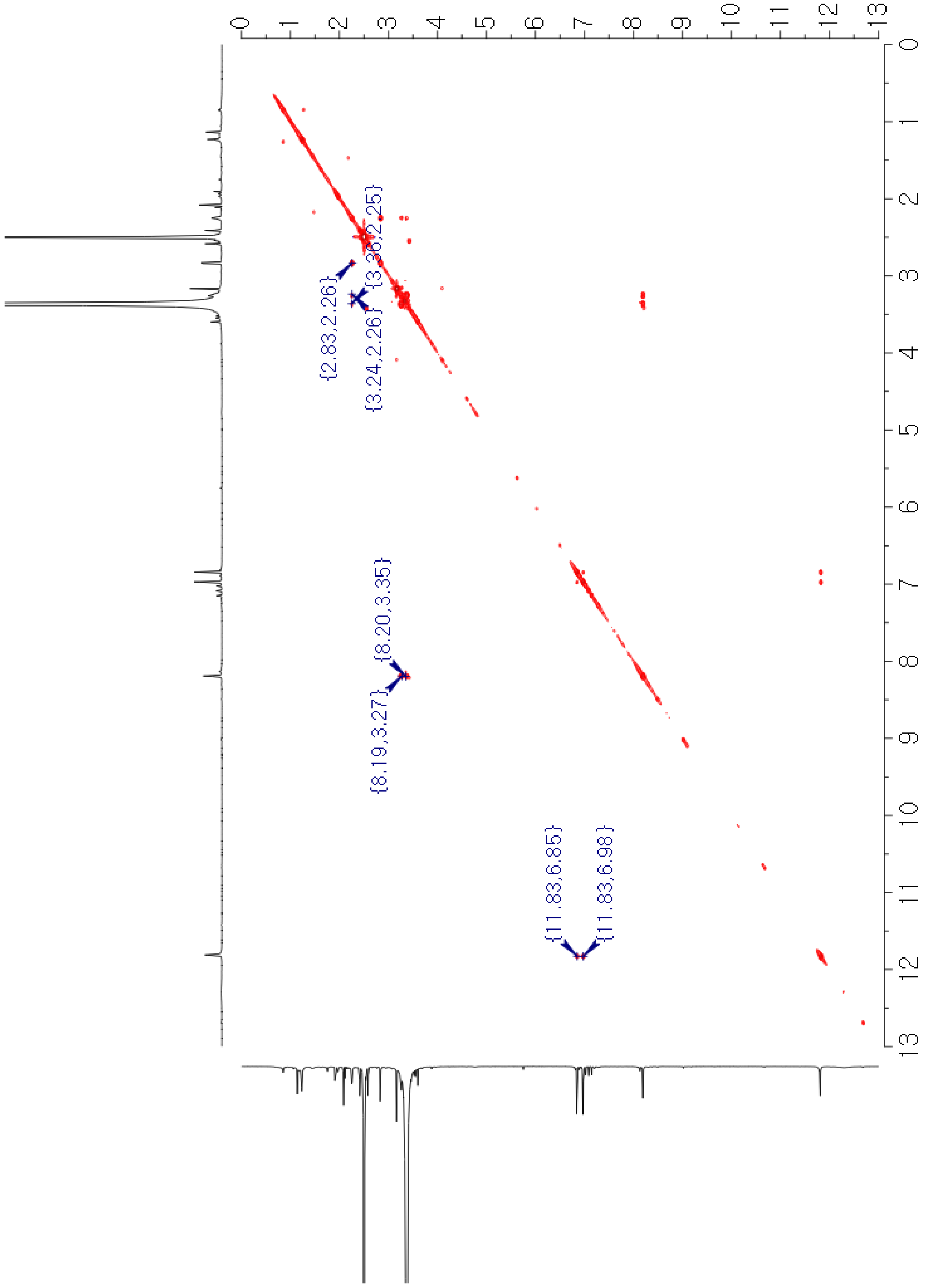

Figure S9. The COSY (600 MHz, DMSO-d_6) spectrum of **2**

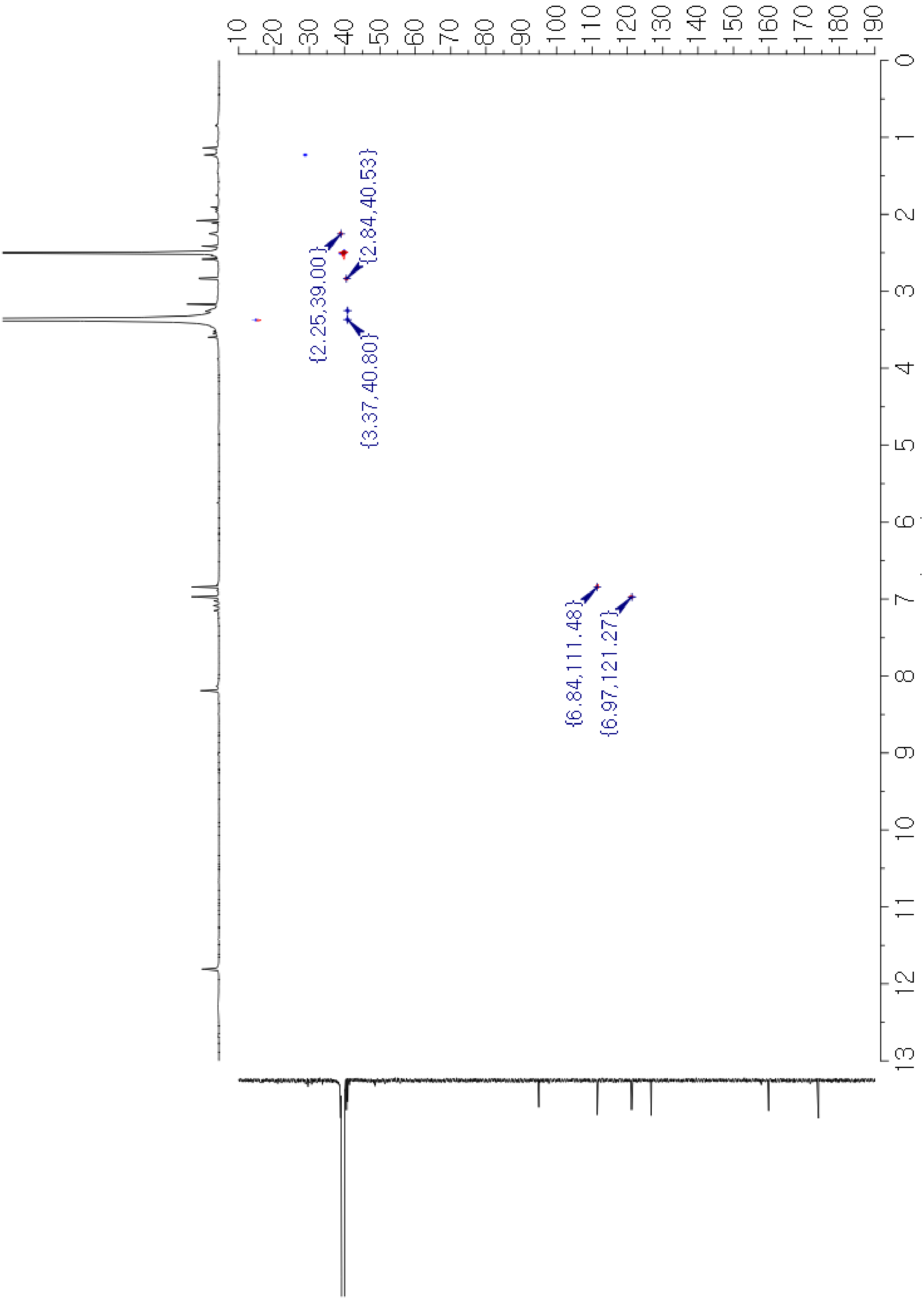

Figure S10. The eHSQC (600 MHz, DMSO-d_6) spectrum of **2**

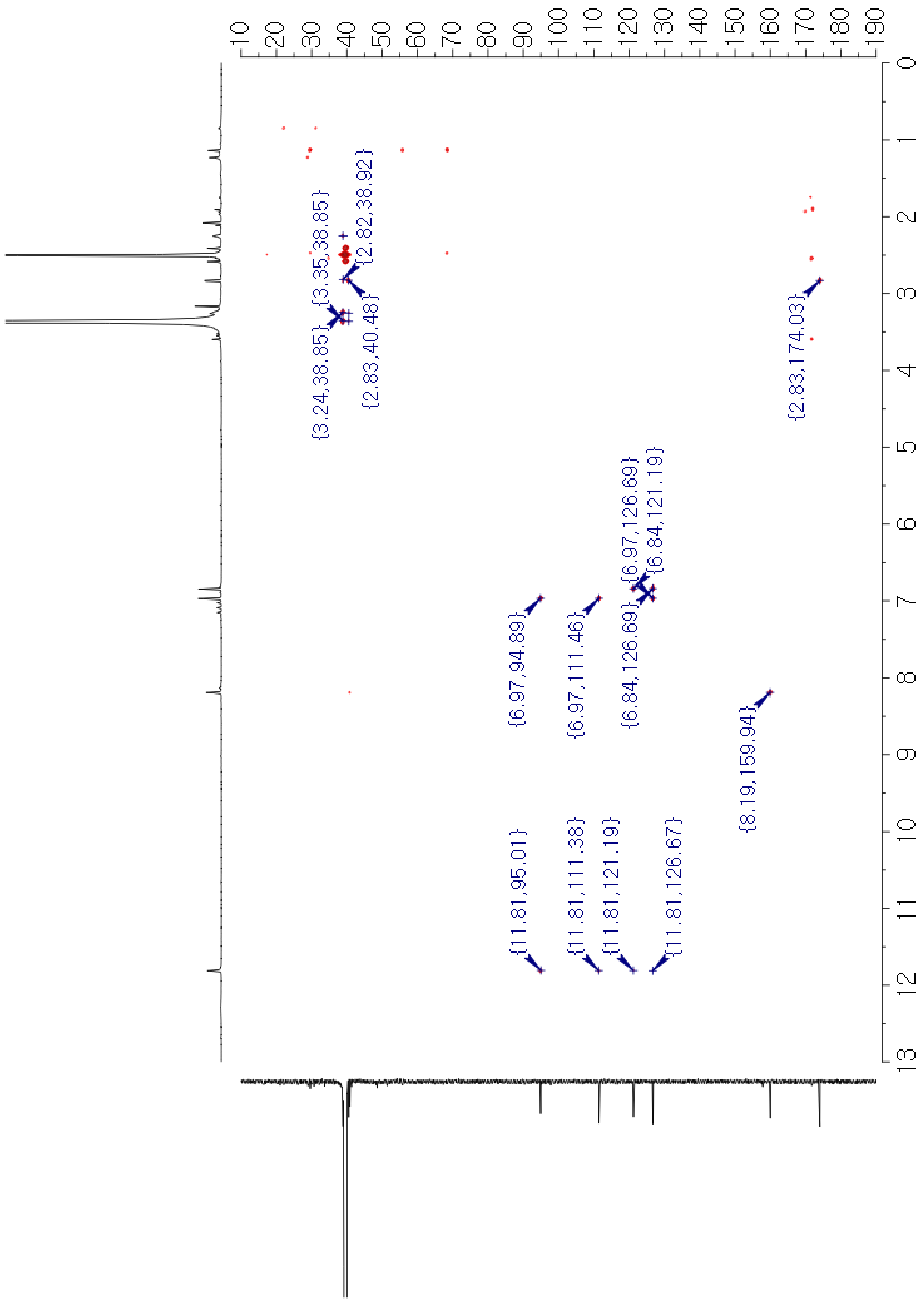

Figure S11. The HMBC (600 MHz, DMSO-d_6) spectrum of **2**

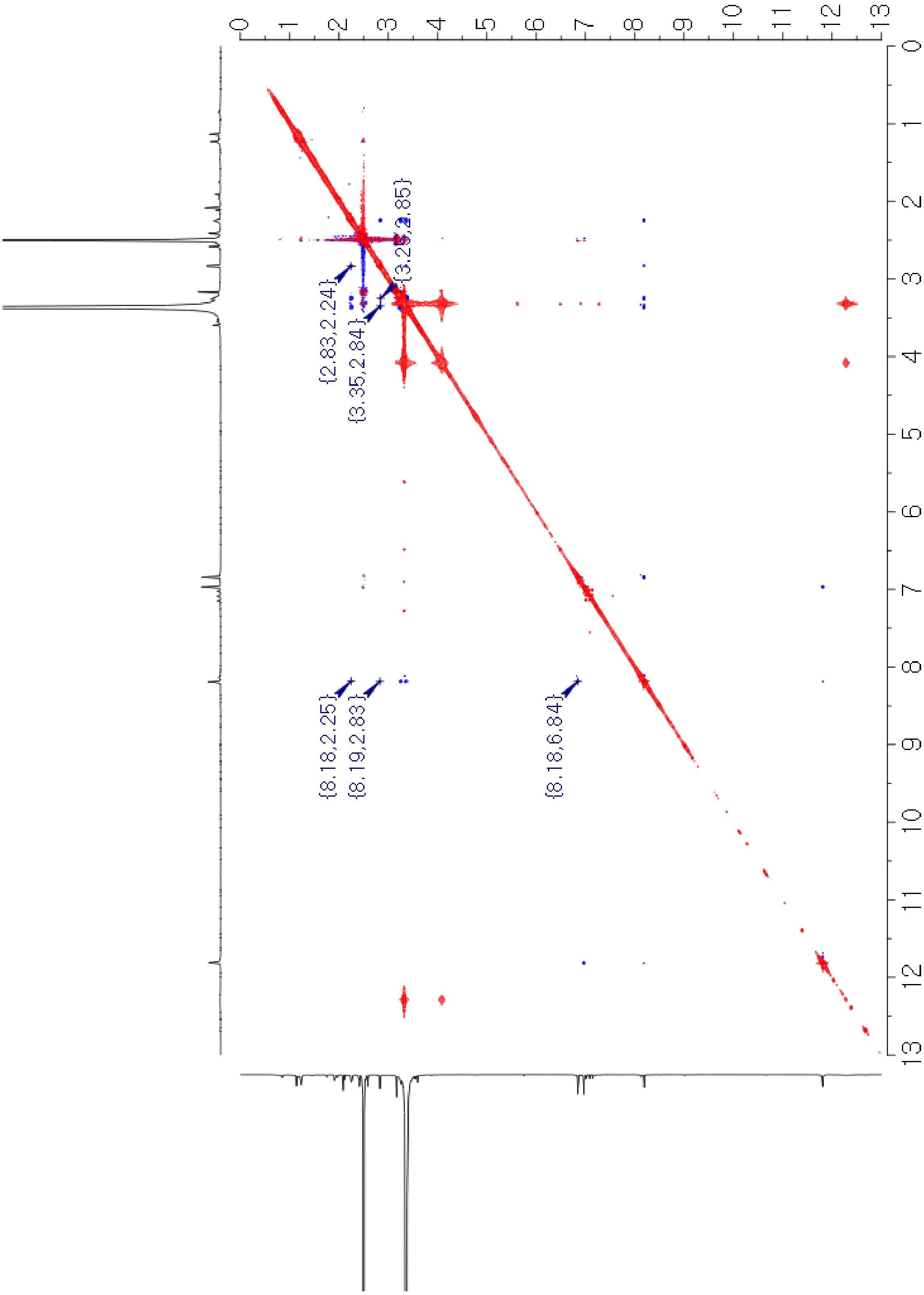

Figure S12. The ROESY (600 MHz, DMSO-d_6) spectrum of **2**

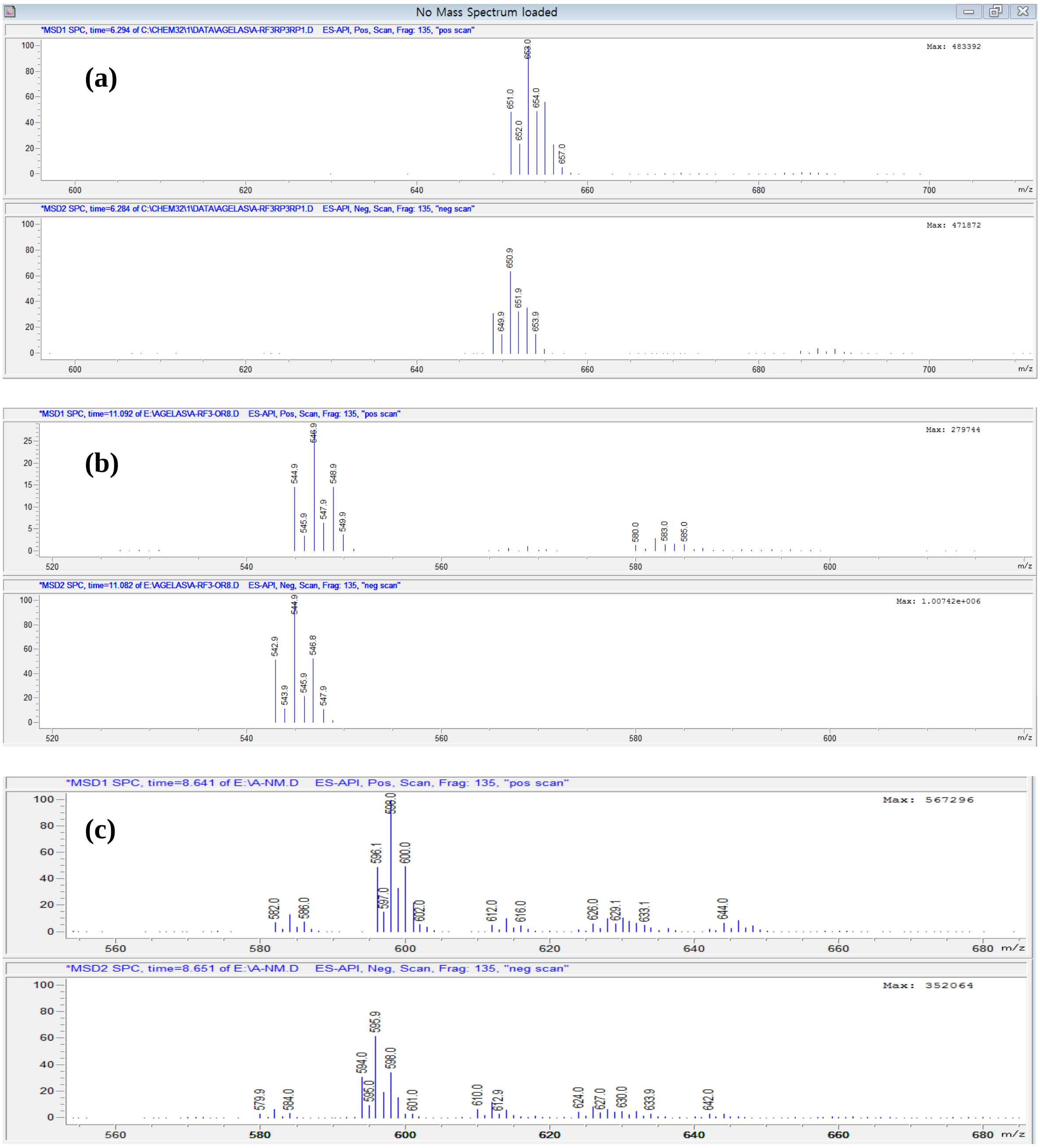

Figure S13. ESI/MS isotopic cluster patterns of compounds **1**, **2** and **6** ((a)-(c)) in positive and negative ion modes

(a)

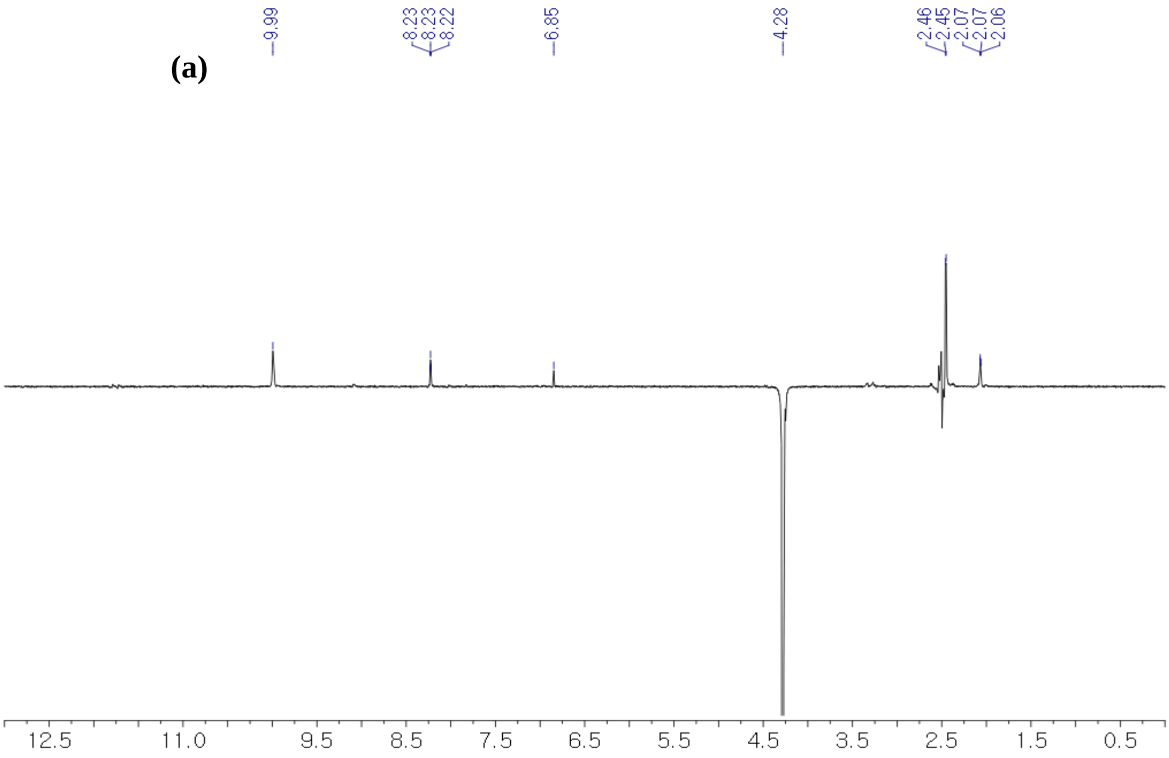
9.99
8.23
8.23
8.22
6.85
4.28
2.46
2.45
2.07
2.07
2.06
12.5
11.0
9.5
8.5
7.5
6.5
5.5
4.5
3.5
2.5
1.5
0.5

(b)

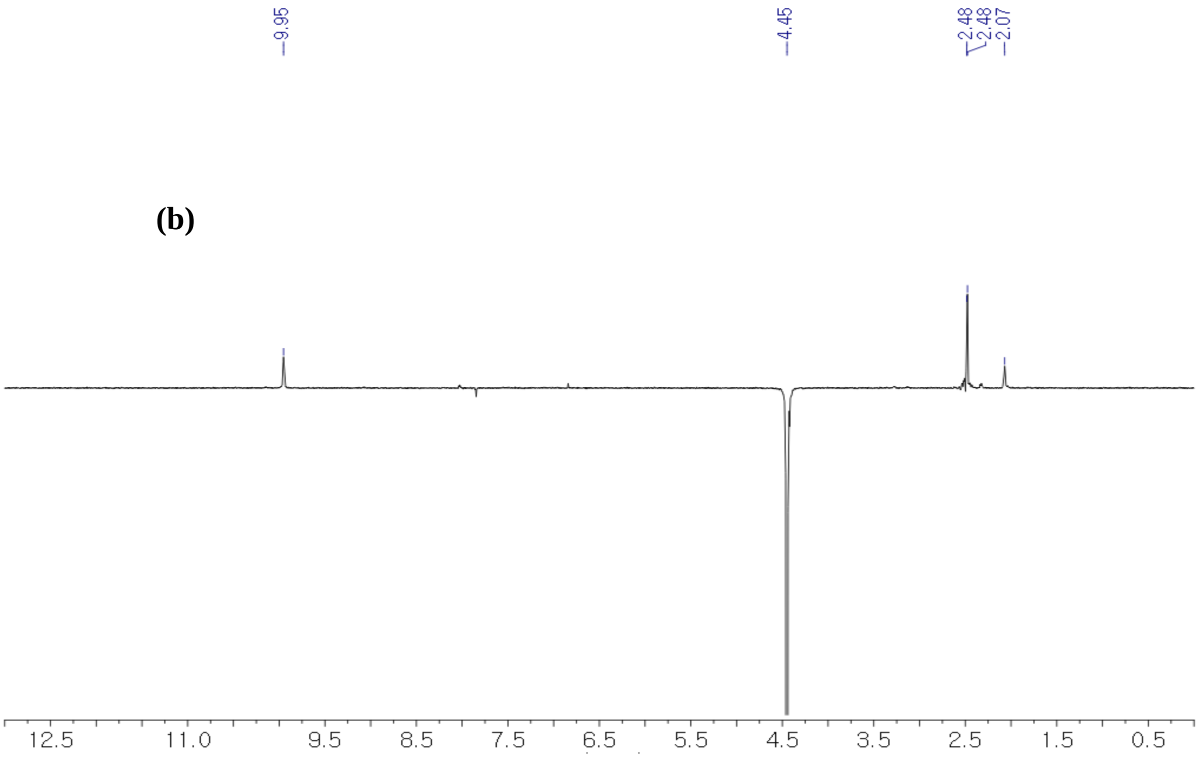
9.95
4.45
2.48
2.48
2.07
12.5
11.0
9.5
8.5
7.5
6.5
5.5
4.5
3.5
2.5
1.5
0.5

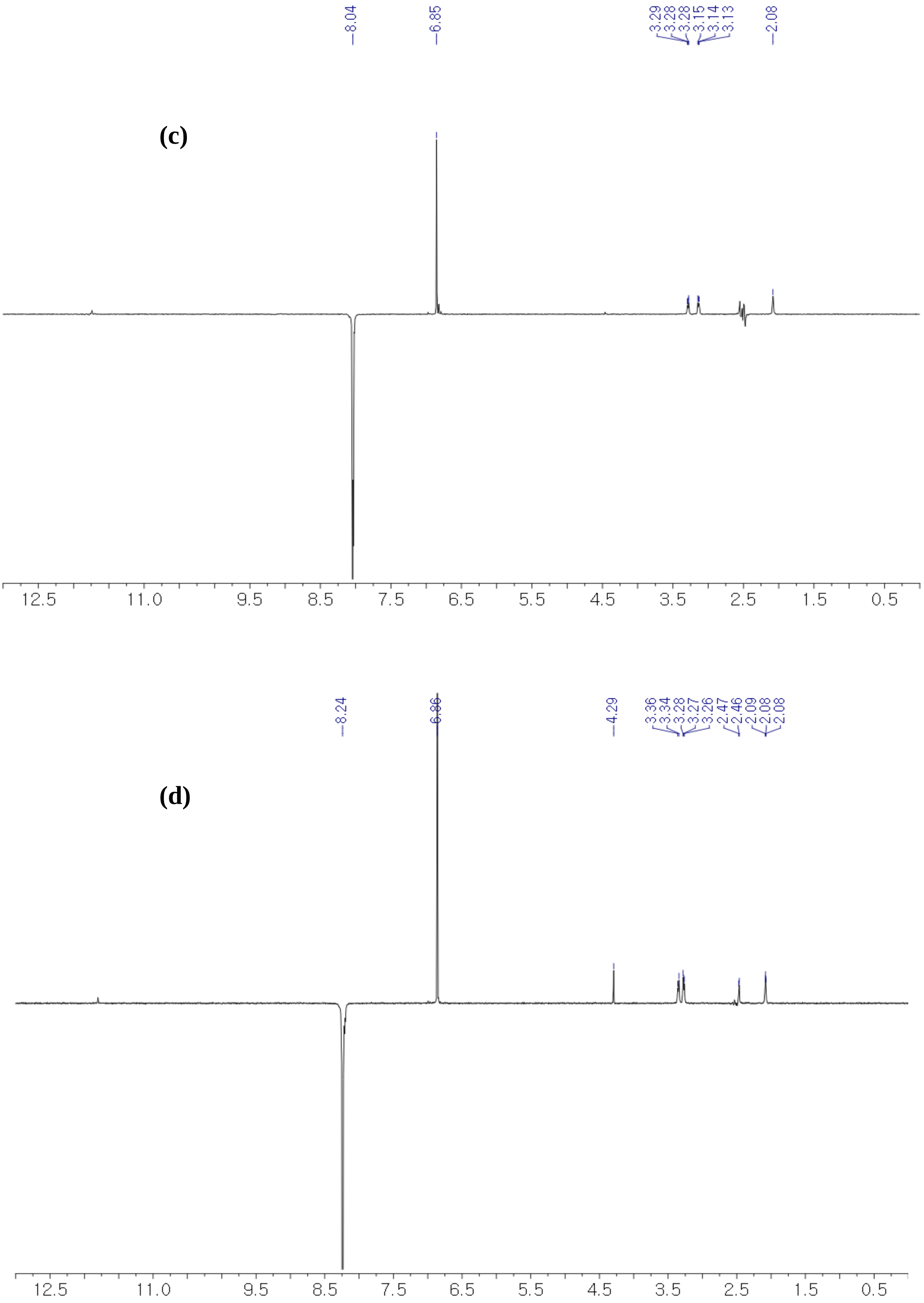

Figure S14. 1D selective gradient ROESY experiments (600 MHz, DMSO-d_6) of **1**: Irradiation of the protons at δ_H 4.28, 4.45, 8.04, and 8.24 for (a)-(d), respectively.

(a)

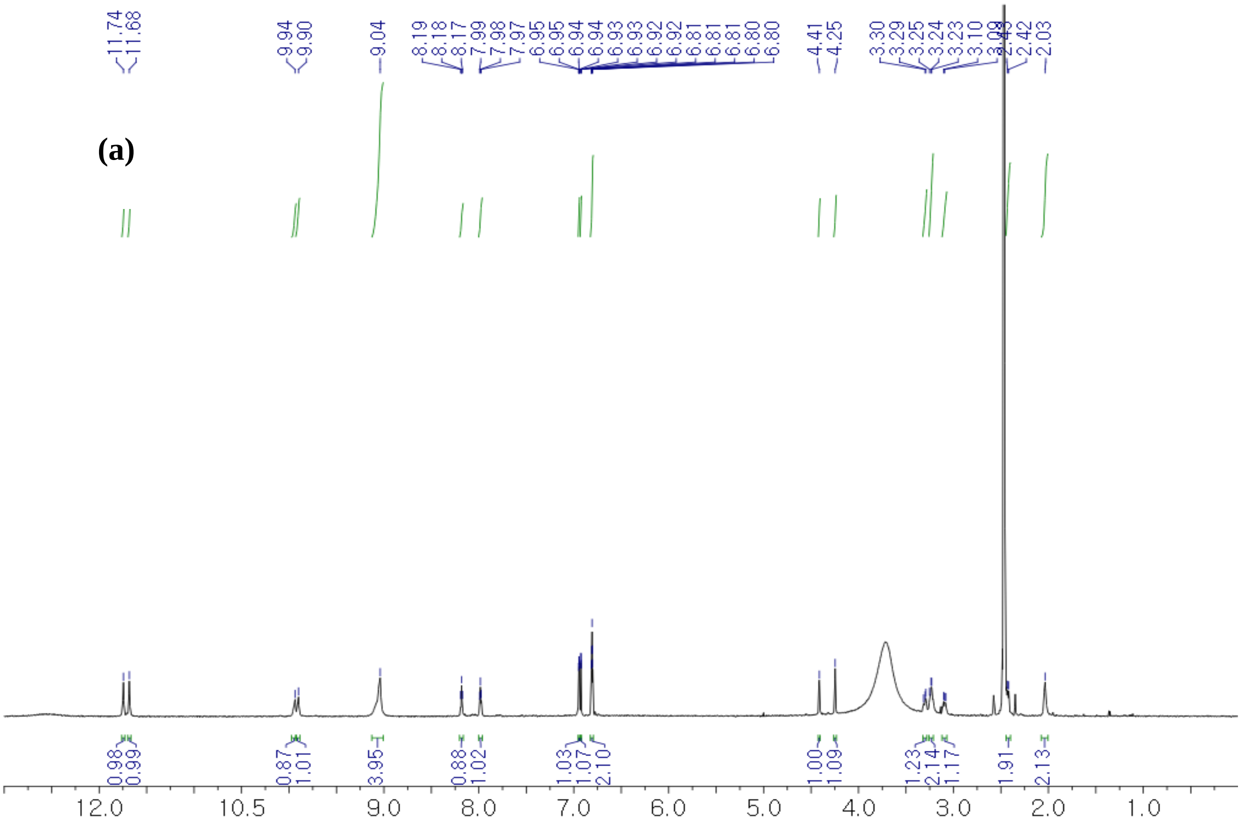

(b)

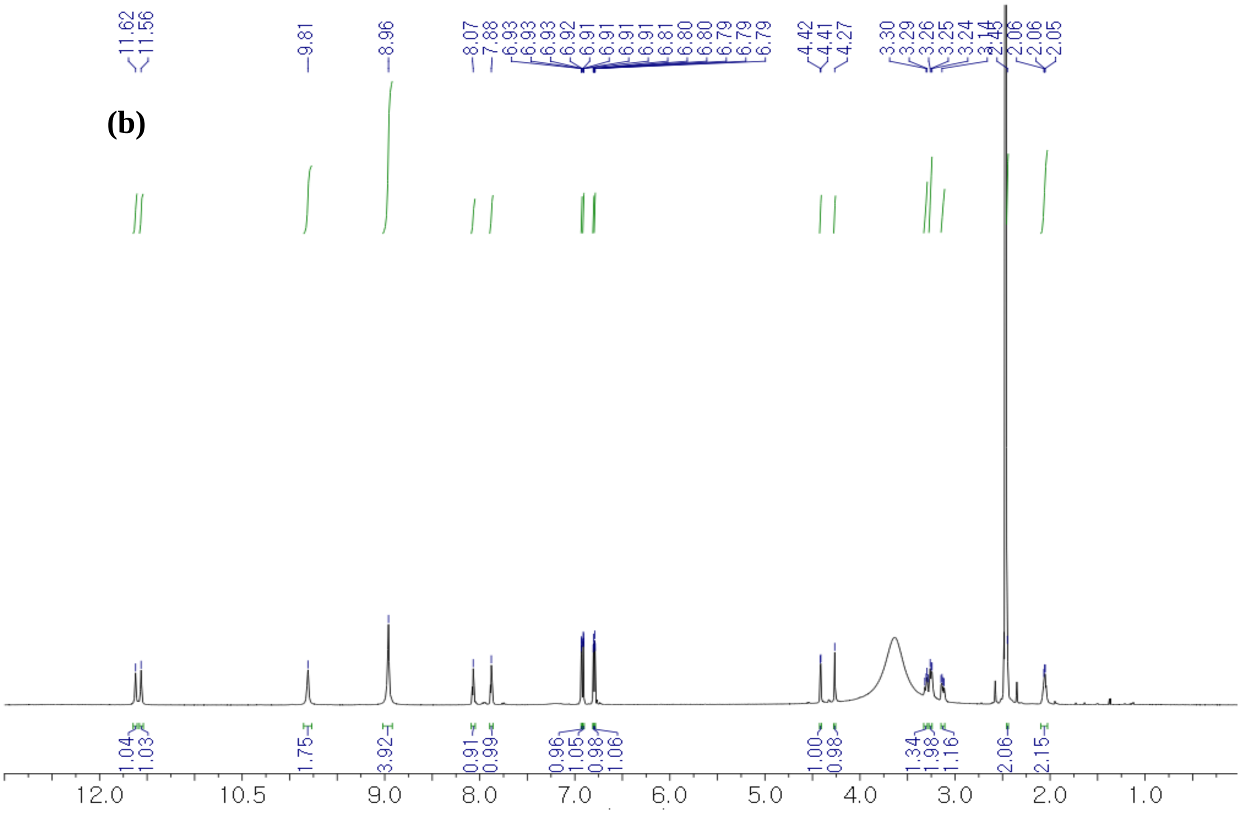

(c)

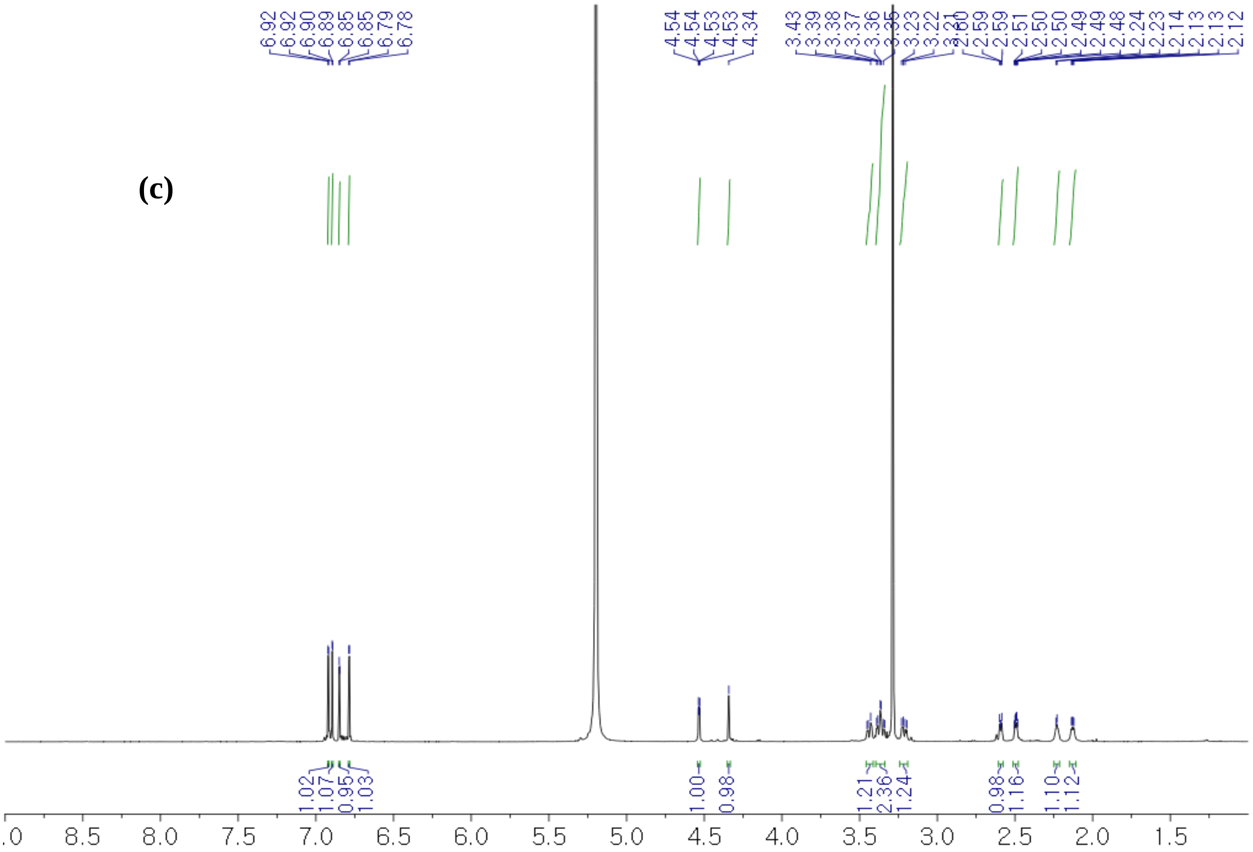

(d)

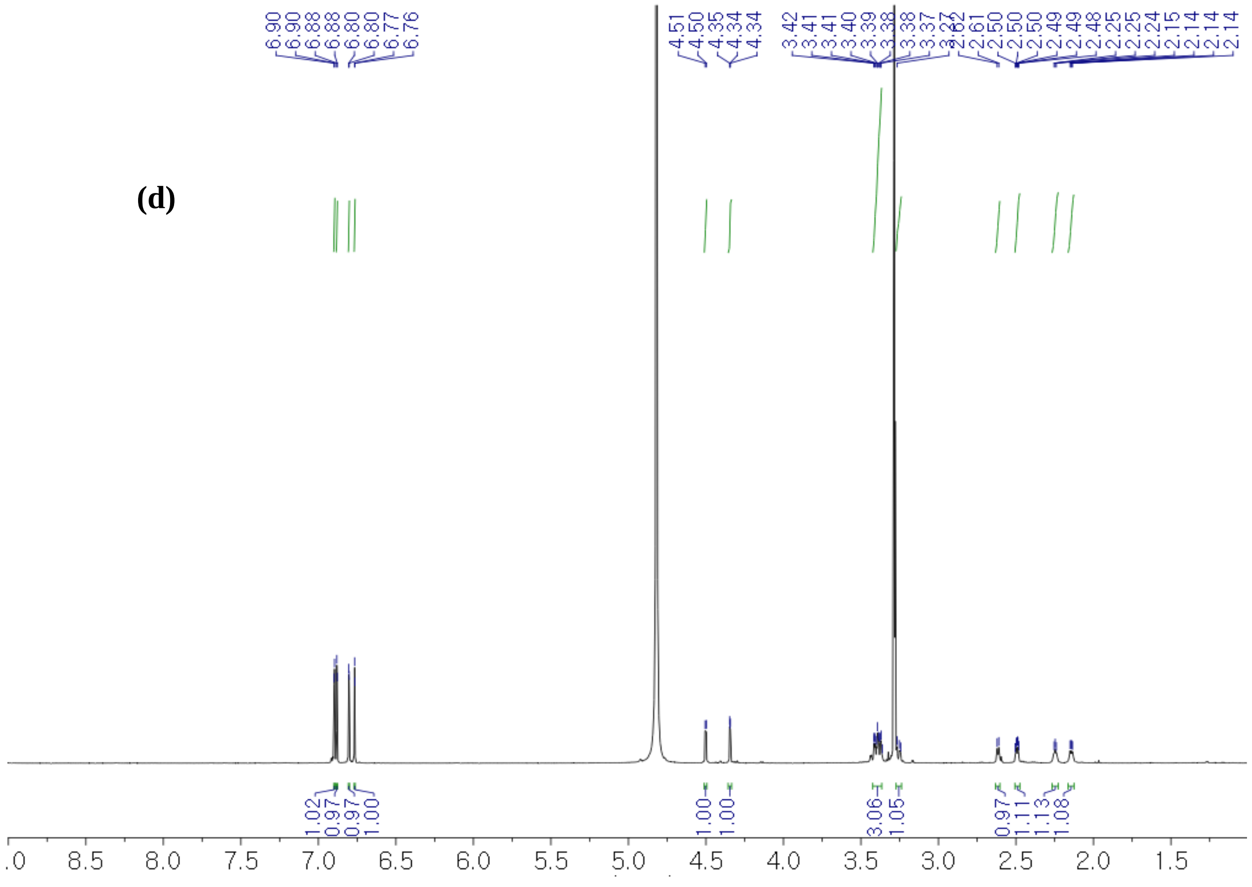

(e)

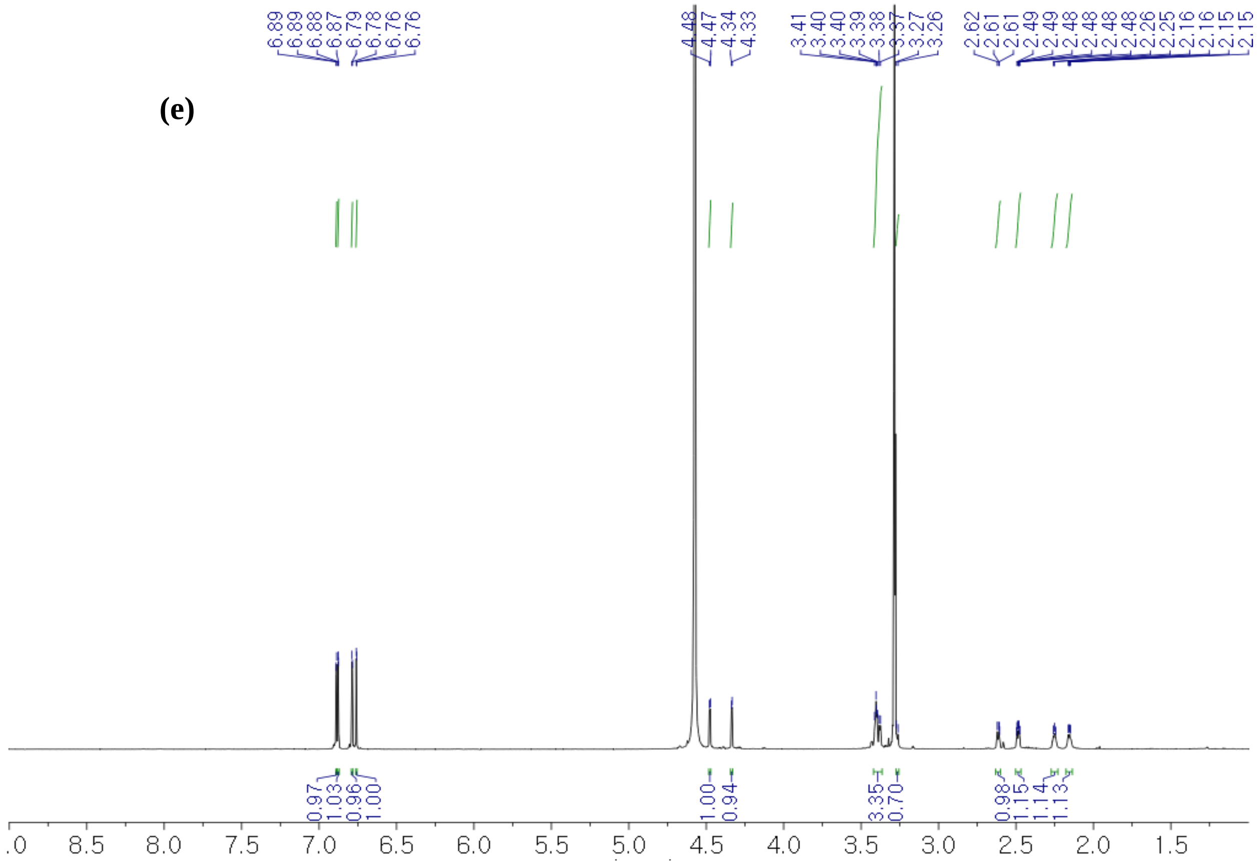

Figure S15. The variable temperature NMR experiments (DMSO-d_6) of **1**: (a) +25 ʹC and (b) +50 ʹC (MeOH-d_4) of **1**: (c) -15 ʹC, (d) +25 ʹC, and (e) +50 ʹC

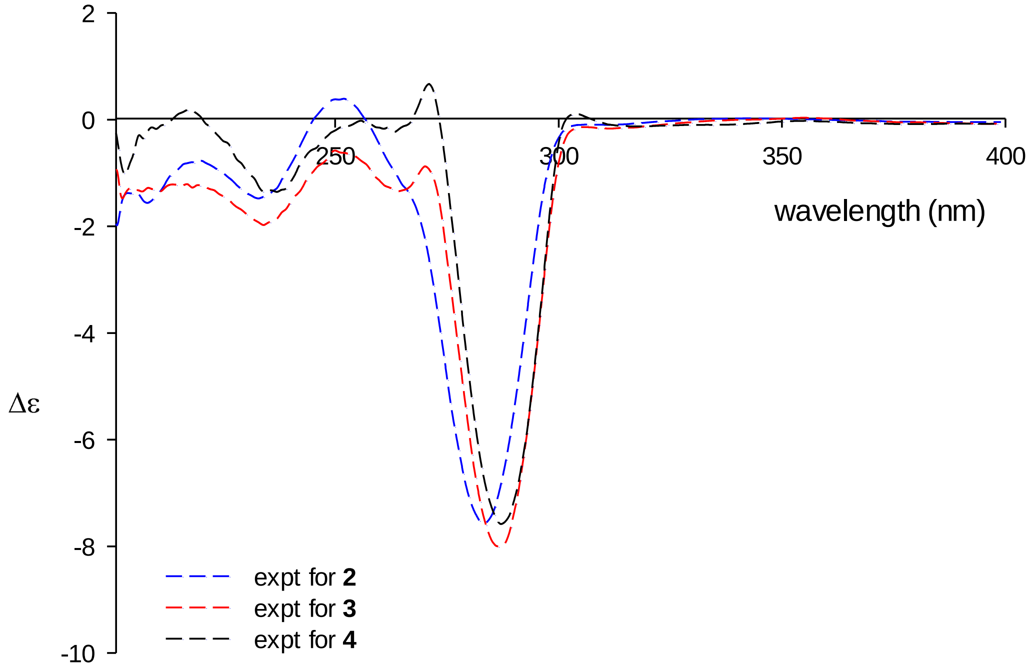

Figure S16. The CD spectra of **2-4**

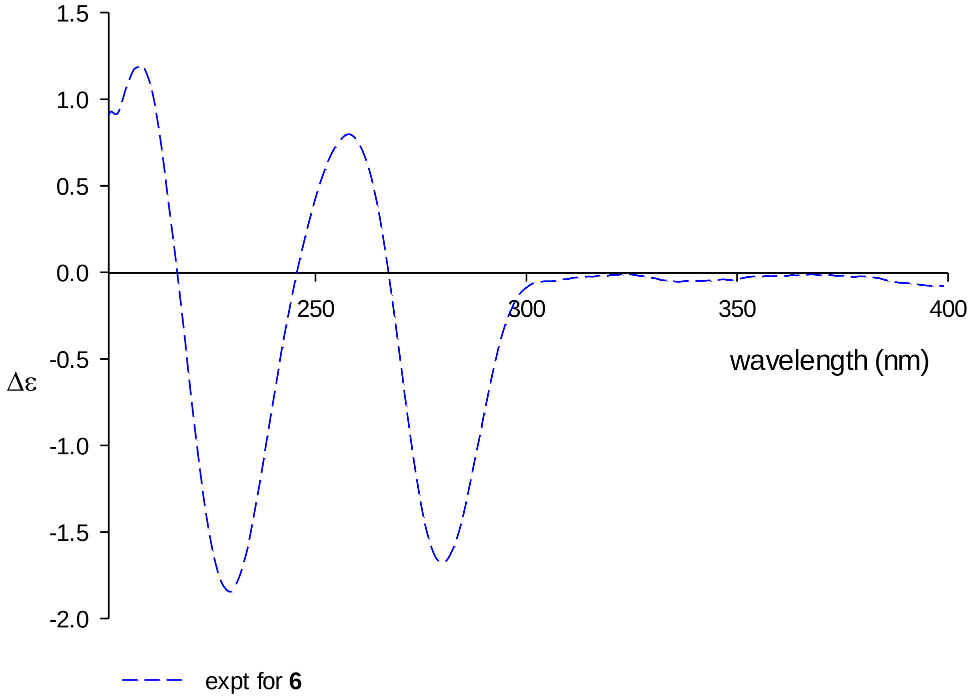

Figure S17. The CD spectrum of **6**
